# Genomic Evidence for Simultaneous Optimization of Transcription and Translation through Codon Variants in the *pmoCAB* Operon of Type Ia Methanotrophs

**DOI:** 10.1128/mSystems.00342-19

**Published:** 2019-07-23

**Authors:** Juan C. Villada, Maria F. Duran, Patrick K. H. Lee

**Affiliations:** a School of Energy and Environment, City University of Hong Kong, Kowloon, Hong Kong SAR, China; University of Tennessee at Knoxville

**Keywords:** codon usage, methane monooxygenase, resource allocation, synthesis cost, translation efficiency

## Abstract

Understanding the interplay between genotype and phenotype is a fundamental goal of functional genomics. Methane oxidation is a microbial phenotype with global-scale significance as part of the carbon biogeochemical cycle and a sink for greenhouse gas. Microorganisms that oxidize methane (methanotrophs) are taxonomically diverse and widespread around the globe. In methanotrophic bacteria, enzymes in the methane oxidation metabolic module (KEGG module M00174, conversion of methane to formaldehyde) are encoded in four operons (*pmoCAB*, *mmoXYZBCD*, *mxaFI*, and *xoxF*). Recent reports have suggested that methanotrophs in *Proteobacteria* acquired methane monooxygenases through horizontal gene transfer. Here, we used a genomic meta-analysis to infer the transcriptional and translational advantages of coding sequences from the methane oxidation metabolic modules of different types of methanotrophs. By analyzing isolate and metagenome-assembled genomes from phylogenetically and geographically diverse sources, we detected an anomalous nucleotide composition bias in the coding sequences of particulate methane monooxygenase genes (*pmoCAB*) from type Ia methanotrophs. We found that this nucleotide bias increases the level of codon bias by decreasing the GC content in the third base of codons, a strategy that contrasts with that of other coding sequences in the module. Further codon usage analyses uncovered that codon variants of the type Ia *pmoCAB* coding sequences deviate from the genomic signature to match ribosomal protein-coding sequences. Subsequently, computation of transcription and translation metrics revealed that the *pmoCAB* coding sequences of type Ia methanotrophs optimize the usage of codon variants to maximize translation efficiency and accuracy, while minimizing the synthesis cost of transcripts and proteins.

**IMPORTANCE** Microbial methane oxidation plays a fundamental role in the biogeochemical cycle of Earth’s system. Recent reports have provided evidence for the acquisition of methane monooxygenases by horizontal gene transfer in methane-oxidizing bacteria from different environments, but how evolution has shaped the coding sequences to execute methanotrophy efficiently remains unexplored. In this work, we provide genomic evidence that among the different types of methanotrophs, type Ia methanotrophs possess a unique coding sequence of the *pmoCAB* operon that is under positive selection for optimal resource allocation and efficient synthesis of transcripts and proteins. This adaptive trait possibly enables type Ia methanotrophs to respond robustly to fluctuating methane availability and explains their global prevalence.

## Abstract (precis)

Microbial methane oxidation plays a fundamental role in the biogeochemical cycle of Earth’s system. Recent reports have provided evidence for the acquisition of methane monooxygenases by horizontal gene transfer in methane-oxidizing bacteria from different environments, but how evolution has shaped the coding sequences to execute methanotrophy efficiently remains unexplored. In this work, we provide genomic evidence that among the different types of methanotrophs, type Ia methanotrophs possess a unique coding sequence of the *pmoCAB* operon that is under positive selection for optimal resource allocation and efficient synthesis of transcripts and proteins. This adaptive trait possibly enables type Ia methanotrophs to respond robustly to fluctuating methane availability and explains their global prevalence.

## INTRODUCTION

Microbial methane oxidation plays a number of fundamental roles in the global ecosystem (1). Methane-oxidizing microorganisms can mitigate methane emissions by acting as methane sinks (2, 3) and thereby reduce the contribution of methane to climate change (4). Microbial oxidation of methane also provides an entry point for methane into the global food web and can serve as a primary carbon source for large trophic systems (5, 6). Methane oxidation is a globally distributed phenotype expressed in microorganisms from diverse taxonomic groups. Based on a range of phenotypic (e.g., metabolic pathways for carbon fixation, fatty acid compositions) and phylogenetic (e.g., *Proteobacteria*, *Verrucomicrobia*, NC10) features, methanotrophic bacteria can be categorized into seven major types (7): Ia, Ib, Ic, IIa, IIb, III, and the candidate division NC10. The distribution of marker gene sequences for the major methanotroph types suggests that they are differentially prevalent across environments (7). In bacteria, methane oxidation begins with two enzymatic steps where methane is first converted to methanol by a soluble or particulate methane monooxygenase (sMMO or pMMO, respectively), and then methanol is oxidized to formaldehyde by a pyrroloquinoline quinone-containing methanol dehydrogenase that can be calcium dependent (Mxa) or lanthanide dependent (Xox). The resulting formaldehyde can be directed to energy production or biomass synthesis (8). The functional sMMO is encoded in the six-gene operon *mmoXYZBCD*, while pMMO is encoded in the three-gene operon *pmoCAB*. Of the methanol dehydrogenases, the functional Mxa enzyme is encoded in the two-gene operon *mxaFI* and Xox is encoded by the gene *xoxF*.

In evolutionary history, methane oxidation appeared at around the same time as oxygenic photosynthesis, nitrogen fixation, nitrification, and denitrification (9), and it is possible that the emergence of methanotrophy occurred soon after the last universal common ancestor (10). Hence, evolution has likely shaped methanotrophs, with many as-yet-undiscovered properties (10). One unexplored question of fundamental importance to our understanding of methanotrophy is how the genes that encode the methane oxidation metabolic module have been shaped by evolution to efficiently execute methanotrophy, especially after recent reports have suggested that MMOs were potentially acquired by horizontal gene transfer in some types of methanotrophs (11–14). In the three cellular domains and in viruses, evolution has selected gene sequences that perform cellular functions beyond just encoding the amino acid compositions of proteins. For example, gene sequences have nucleotide and codon variants that direct mRNA folding (15, 16), transcript abundance (17), mRNA degradation (18), RNA toxicity (19), protein synthesis (20), and cotranslational protein folding (21), and they promote the interaction of peptides with the signal recognition particle (22). Gene sequences can also affect the cellular economy of protein synthesis (23), reduce the metabolic burden of nucleotide synthesis by incorporating less expensive nucleotides (24), and allocate resources required for transcription (25) and translation (26).

In this study, we performed an in-depth genomic meta-analysis to investigate the coding sequences of the genes that encode the methane oxidation metabolic module (as defined in the KEGG metabolic module M00174 [conversion of methane to formaldehyde]) in bacteria. We found that evolution has shaped the *pmoCAB* operon of type Ia methanotrophs with a unique coding sequence that optimizes resource allocation by reducing the biosynthetic costs of transcription and translation while ensuring translation efficiency and accuracy. This study provides novel insights into the molecular biology and evolution of methanotrophic bacteria and extends our understanding of the mechanisms developed by nature to sustain metabolism and life on Earth.

## RESULTS

### Meta-analysis of methanotroph genomes.

To investigate the coding sequences of genes encoding the methane oxidation metabolic module, we analyzed 59 methanotrophic bacterial genomes, including 47 isolate genomes and 12 metagenome-assembled genomes (MAGs) (see Fig. S1 and Table S1 in the supplemental material). These genomes originated from different sampling locations around the globe (Fig. S1a) and were phylogenetically diverse (Fig. S1b), comprising 17 genera from five families and with GC contents ranging from 40% (Methylacidiphilum kamchatkensis Kam1) to 65% (Methylosinus trichosporium OB3b). The 59 methanotroph genomes were categorized into five major types (7): Ia (*n *=* *34), Ib (*n *=* *6), IIa (*n *=* *11), IIb (*n *=* *5), and III (*n *=* *3).

10.1128/mSystems.00342-19.1 FIG S1 Nucleotide composition biases of genomes. (a) Genomes analyzed in this study were geographically mapped to the locations where the samples originated. (b) A phylogenetic tree was reconstructed based on whole-genome sequences. The total number of coding sequences (*n*) used to calculate the statistics of the GC and GC_3_ contents for each organism is indicated. (c) Extended analysis of the nucleotide composition biases of all coding sequences in the methane oxidation metabolic module and outgroups (*hao* and *pxmABC*) in all methanotrophs. The dashed line indicates equality between the GC and GC_3_ contents. The shaded region below the equality line shows the data points where the GC_3_ content is less than the GC content. Download FIG S1, PDF file, 2.1 MB. Copyright © 2019 Villada et al. 2019 Villada et al. This content is distributed under the terms of the Creative Commons Attribution 4.0 International license.

10.1128/mSystems.00342-19.8 TABLE S1 Detailed information and sources of the 59 genomes of methanotrophic bacteria, including the 47 isolate genomes and 12 MAGs used in this study. Download Table S1, XLSX file, 0.03 MB. Copyright © 2019 Villada et al. 2019 Villada et al. This content is distributed under the terms of the Creative Commons Attribution 4.0 International license.

### Type Ia *pmoCAB* coding sequences have an anomalous nucleotide composition bias.

**FIG 1:**
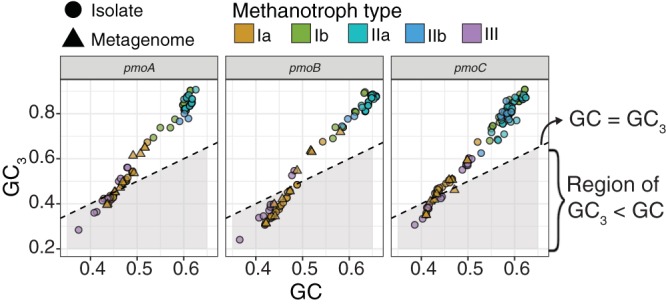
Nucleotide composition bias of *pmoCAB* coding sequences. An extended analysis of all coding sequences is shown in Fig. S1c.

We measured the distribution of nucleotide composition biases of all coding sequences in each genome (Fig. S1b). The guanine-plus-cytosine (GC) content of every coding sequence was calculated, as well as the GC content in the third nucleotide of each codon (GC_3_ content). In genomes of methanotroph types Ia, Ib, IIa, and IIb, the GC_3_ contents of coding sequences tended to be higher than their GC contents, while in type III genomes, GC_3_ contents tended to be lower than GC contents (Fig. S1b). Next, we analyzed the nucleotide composition biases of the 12 coding sequences in the four operons forming the methane oxidation metabolic module (KEGG M00174, methane to formaldehyde) (Fig. S1c). All coding sequences from all five types showed patterns of relative GC/GC_3_ bias consistent with those of the whole genomes (Fig. S1), except for *pmoCAB* coding sequences of type Ia methanotrophs, where the GC_3_ content was lower than the GC content (Fig. 1). The *pxmABC* and *hao* coding sequences, encoding a copper‐containing membrane monooxygenase (13) and a hydroxylamine oxidoreductase (27), respectively, were selected as outgroups as they are present in a large number of methanotroph genomes but are not part of the set of genes that formed the focus of this study. As expected, these outgroup coding sequences showed a pattern of relative GC/GC_3_ bias consistent with those of the whole genomes (Fig. S1c). As with the type Ia *pmoCAB* sequences, the type III coding sequences tended to have lower GC_3_ contents than the GC contents (Fig. S1c), consistent with the whole-genome pattern in type III genomes (Fig. S1).

### The anomalous nucleotide composition of type Ia *pmoCAB* coding sequences provides an atypical codon usage bias.

The anomalous nucleotide composition of type Ia *pmoCAB* coding sequences led us to hypothesize that this may generate an atypical bias in codon usage. To test whether codon usages differ among coding sequences in the methane oxidation metabolic module of each methanotroph, we first calculated the relative synonymous codon usage (RSCU) in every coding sequence (outgroups were also included). On ordination of the RSCU values of type Ia coding sequences (Fig. S2a), *pmoCAB* coding sequences clustered separately (Euclidean distance permutational multivariate analysis of variance [PERMANOVA], *F* = 36.85, *P < *0.01), and the effect of the source operon in the separation was strong (*R*^2^ = 0.38). A reduced effect of the source operon was observed in the clustering pattern for coding sequences of types Ib (*F* = 3.82, *P < *0.01, *R*^2^ = 0.21) and IIa (*F* = 12.67, *P < *0.01, *R*^2^ = 0.24), while weak effects (*R*^2^ < 0.2) or nonsignificant probabilities (*P ≥ *0.01) were observed for types IIb and III (Fig. S2).

10.1128/mSystems.00342-19.2 FIG S2 Extended analysis of the RSCU biases of all coding sequences in the methane oxidation metabolic module and outgroups (*hao* and *pxmABC*) in all methanotrophs. Principal-component analysis (PCA) of the RSCU values for the four coding sequences of the methane oxidation metabolic module and two outgroups of type Ia (a), type Ib (b), type IIa (c), type IIb (d), and type III (e) methanotrophs. Plots at the right-hand side of each PCA show the varied (codon) loadings of the PCA. Download FIG S2, PDF file, 1.5 MB. Copyright © 2019 Villada et al. 2019 Villada et al. This content is distributed under the terms of the Creative Commons Attribution 4.0 International license.

**FIG 2:**
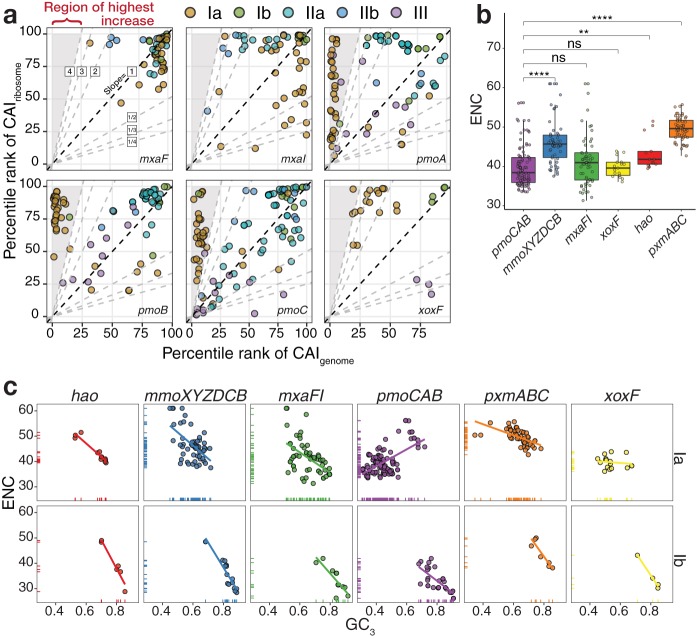
Analysis of the codon usage bias. (a) Analysis of the codon adaptation index (CAI). CAI indexes were converted to percentile ranks based on the relative distribution of CAIs in each methanotroph. Dashed lines with various slopes delineate the variations between the percentile ranks of both indexes. (b) Analysis of the distribution of the effective numbers of codons (ENC) of coding sequences from type Ia methanotrophs. The two-tailed Wilcoxon signed-rank test was applied to test the difference of ENC values between the *pmoCAB* and other coding sequences. A nonsignificant result of the tests is denoted by ns (*P > *0.01). **, *P ≤ *0.01; ***, *P ≤ *0.001; ****, *P ≤ *0.0001. (c) Analysis of the ENC as a function of GC_3_ content, with the line representing the fit of a linear model between the ENC and the GC_3_ content.

This result indicates that the type Ia *pmoCAB* coding sequences have an atypical codon usage bias compared to the codon usages of other coding sequences in the methanotrophy module. We hypothesized that this may be an adaptation to allow for higher expression of these genes. To test this, we calculated a codon adaptation index (CAI) that compared the codon usage of a coding sequence to the codon usage of a reference set of sequences from the same genome. The first CAI, named CAI_genome_, used the codon frequency of all coding sequences in the genome as a reference, while the second CAI, named CAI_ribosome_, used the codon frequency of only ribosomal protein genes as a reference. The CAI_ribosome_ thus served as a proxy to represent the codon usage of highly expressed genes. The comparison between the CAI_genome_ and CAI_ribosome_ showed that *mxaF* coding sequences tended to have codon usages similar to those of both reference sets in all types of methanotrophs (Fig. 2a), which was also observed for *mxaI* and *xoxF* coding sequences in type Ib genomes. Type Ia *mxaI* coding sequences tended to deviate from the codon bias of ribosomal protein genes and toward the genome-scale codon usage pattern, while the opposite was observed for type IIa and IIb *mxaI* coding sequences. Type Ia *xoxF* coding sequences appeared in the top percentile ranks of the CAI_ribosome_ but below the 50th percentile ranks of the CAI_genome_. A similar but much stronger pattern was observed in type Ia *pmoCAB* coding sequences, which appeared in the bottom 10% of CAI_genome_ values but in much higher percentile ranks for the CAI_ribosome_ (Fig. 2a). The results for all coding sequences in the methane oxidation module and outgroups are given in Fig. S3a. Overall, the extreme deviation of type Ia *pmoCAB* coding sequences from the genomic norm shows that they have a codon usage more similar to that of the ribosomal protein-coding sequences.

10.1128/mSystems.00342-19.3 FIG S3 Extended analysis of the CAI and ENC. (a) Coding sequences in the methane oxidation metabolic module and outgroups (*hao* and *pxmABC*) in all methanotrophs. CAIs were converted to percentile ranks based on the relative distribution of CAIs in each methanotroph. Dashed lines with various slopes delineate the variations between the percentile ranks of both indexes. (b) Comparison of the ENCs of coding sequences of *pmoCAB*, ribosomal proteins, and whole genomes of type Ia methanotrophs. The two-tailed Wilcoxon signed-rank test with a 99% confidence level was used to test the significance of the difference. ****, *P ≤ *0.0001. (c) Extended analysis of the ENC as a function of the GC_3_ contents of all coding sequences in the methane oxidation metabolic module and outgroups (*hao* and *pxmABC*) in all methanotrophs. The line represents the fit of a linear model with the formula ENC ∼ GC_3_ content. Download FIG S3, PDF file, 1.4 MB. Copyright © 2019 Villada et al. 2019 Villada et al. This content is distributed under the terms of the Creative Commons Attribution 4.0 International license.

This deviation of codon usage from the genomic signature may be due to a higher codon usage bias in *pmoCAB* coding sequences. To test this, we computed the effective number of codons (ENC) of every coding sequence in all type Ia methanotrophs. The ENC measures the number of synonymous codons used to encode amino acids. Thus, the ENC is 61 if a coding sequence uses all codons equally to produce all 20 standard amino acids, whereas the ENC is 20 if only one codon is used per amino acid (i.e., it has a high codon usage bias). Contrarily to our hypothesis, the ENC of type Ia *pmoCAB* coding sequences was not significantly different (two-tailed Wilcoxon signed-rank test, 99% confidence level) from those of *mxaFI* and *xoxF* coding sequences (Fig. 2b), while the ENC of *pmoCAB* coding sequences was significantly lower than those of coding sequences of ribosomal proteins and whole genomes (Fig. S3b). Overall, this indicates that even though the codons in coding sequences of *pmoCAB* and ribosomal proteins tend to be similar (Fig. 2a), the codon usage bias of *pmoCAB* coding sequences is higher. We hypothesized that if the type Ia *pmoCAB* coding sequences have a codon usage bias similar to those of other coding sequences in the methane oxidation metabolic module (Fig. 2b) but their codon usage frequencies are divergent (Fig. S2a), there should be a clear association between the level of codon bias (Fig. 2b) and the anomalous nucleotide composition bias (Fig. 1). We found that type Ia *pmoCAB* coding sequences increase their level of codon bias at the expense of decreasing the GC_3_ content (Fig. 2c), which contrasts with what occurs with all other methanotroph types and coding sequences tested (Fig. S3c). The *pmoCAB* coding sequences of type III show patterns similar to those of type Ia, but this is expected based on the whole-genome nucleotide bias (Fig. S1).

### Codon biases in methanotrophy operons optimize translation efficiency.

**FIG 3:**
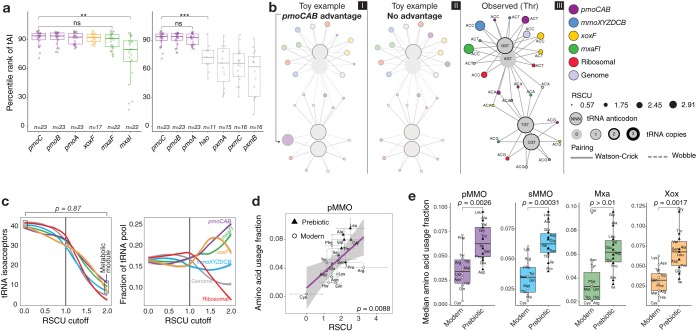
Strategies for optimal translation. (a) The tRNA adaptation index (tAI) was used to estimate the translation efficiencies of coding sequences. tAI values were converted to percentile ranks based on the relative distribution of tAIs in each methanotroph. Coding sequences were ordered by the mean of the tAI percentile rank, and the two-tailed *t* test was applied to test whether the percentile ranks of tAIs are significantly different between the *pmoC* coding sequence (highest mean) and other coding sequence. ns, *P > *0.01; ***, *P ≤ *0.001. (b) Network of the interaction between codons and tRNA in type Ia methanotrophs. The two toy examples illustrate the expected networks when *pmoCAB* coding sequences have a codon usage bias that grants an advantage (marked by an arrow) in the competition for the tRNA pool (I) or does not grant any advantage (II). The observed network for threonine is shown in panel III, and all other amino acids are shown in Fig. S5a. (c) Quantitative analysis of the codon-tRNA interaction network. The figure shows how many copies of tRNA a gene can access according to an RSCU cutoff varying from 0.0 to 2.0, with a step size of 0.01. The reference line at an RSCU of 1.0 indicates the change from the region of nonpreferred codon usage (RSCU < 1.0) to the region of high preference for individual codons (RSCU > 1.0). The colored lines show the fit of a polynomial surface using local fitting. The chi-square test of proportions was applied to test whether the proportions of tRNAs available to coding sequences in the methane oxidation metabolic module are different between an RSCU of 0 and an RSCU of 2. (d) Median usage of each amino acid in the translated coding sequence as a function of the median RSCU value of the codon exhibiting the highest preference for each amino acid for type Ia methanotrophs. The *P* value corresponds to the significance of the linear regression. (e) Usage of prebiotic (inexpensive) and modern (expensive) amino acids in the proteins of the methane oxidation metabolic module of type Ia methanotrophs. The *t* test was used to evaluate the difference between the two groups.

The anomalous nucleotide composition bias of type Ia *pmoCAB* coding sequences suggests that there is a fitness advantage either to the particular codons selected or to having a lower codon diversity. We hypothesized that this advantage may lie in increased translation efficiency. To evaluate this, we first analyzed the encoded tRNA pool of all isolate genomes. MAGs were not included in this analysis, as their tRNA pool might be partial due to incomplete genome assembly. We found that the main difference among methanotrophs is that the type Ia genomes lack tRNAs with cytosine at the 3′ end of the anticodon (Fig. S4a). Taking wobble pairing into account, we queried whether the codon usage bias is an optimization strategy in which type Ia *pmoCAB* codon usage has been selected to better match available tRNA isoacceptors and thereby improve translation efficiency by recruiting the most abundant tRNAs. To address this question, we estimated the translation efficiency of coding sequences using the tRNA adaptation index (tAI). The tAI considers exact and wobble pairing to estimate how efficiently a given coding sequence will be translated based on its codon usage and the genomic tRNA pool. We found that even though the preferences for individual codons are different between type Ia *pmoCAB*, *mxaF*, and *xoxF* coding sequences, their translation efficiencies are similarly ranked in around the top 10th percentile of each genome; the only coding sequence showing a significant difference from *pmoC* is *mxaI* (*t* test, 99% confidence level) (Fig. 3a). We included an analysis to test the difference between the *pmoC* coding sequence and outgroups (Fig. 3a) and found that, in this case, the percentile ranks of tAIs are significantly different (*t* test, 99% confidence level). The tAI results of other coding sequences and methanotrophs can be found in Fig. S4b.

10.1128/mSystems.00342-19.4 FIG S4 (a) tRNA pool of each genome ordered by similarity. (b) Extended analysis of the tAIs of all coding sequences in the methane oxidation metabolic module and outgroups (*hao* and *pxmABC*) in all methanotrophs. tAI values were converted to percentile ranks based on the relative distribution of tAIs in each methanotroph. The black horizontal line represents the median tAI. *n* is the number of coding sequences. (c) Median RSCU values calculated from the distribution of each codon’s RSCU value in all type Ia methanotrophs. (d) Detailed distribution of RSCU values of all coding sequences in the methane oxidation metabolic module and outgroups (*hao* and *pxmABC*) of type Ia methanotrophs. Coding sequences of each type Ia methanotroph are in the columns, and codons are in rows. (e) tRNA pool of each genome. Vertical lines divide the different types of methanotrophs. The type of methanotroph is specified in the labels at the bottom. Download FIG S4, PDF file, 1.1 MB. Copyright © 2019 Villada et al. 2019 Villada et al. This content is distributed under the terms of the Creative Commons Attribution 4.0 International license.

10.1128/mSystems.00342-19.5 FIG S5 (a) Extended network of the interaction between codons and tRNAs in type Ia methanotrophs. The integrated network is composed of median RSCU values, the median number of tRNA copies, and the codon-anticodon pairing rules. The median of the RSCU values was calculated for six sets of coding sequences: four sets in the methane oxidation metabolic module, one set comprising the coding sequences of ribosomal protein genes, and one set comprising all the coding sequences in the genome. (b) Median amino acid usage of each translated coding sequence of type Ia methanotrophs. The vertical black line in each amino acid shows the median of the amino acid composition as the fraction of the total protein composition. (c) Median usage of each amino acid in each translated coding sequence as a function of the median RSCU value of the codon exhibiting the highest preference for each amino acid for type Ia, Ib, IIa, IIb, and III methanotrophs. The *P* value corresponds to the significance of the linear regression. Download FIG S5, PDF file, 1.4 MB. Copyright © 2019 Villada et al. 2019 Villada et al. This content is distributed under the terms of the Creative Commons Attribution 4.0 International license.

Overall, these results suggest that for type Ia methanotrophs, the translation efficiencies of *pmoCAB*, *mxaF*, and *xoxF* coding sequences are similarly high (tAIs ≥ 90th percentile rank) (Fig. 3a), and these coding sequences have been optimized in parallel through different codon usage biases. A possible evolutionary explanation for these observations is that the distinct codon usage bias of each coding sequence causes these genes to segregate between different tRNA isoacceptors, allowing simultaneous translation of the coding sequences required for methane oxidation while avoiding competition for the same tRNAs. To investigate this, we reconstructed the protein synthesis network accounting for the distribution of tRNAs among coding sequences of the metabolic module (Fig. 3b). We also included in the network the median RSCU value of each codon per coding sequence of type Ia methanotrophs (Fig. S4c and d) so that we can account for the codon preference and its interaction with tRNAs through different pairing mechanisms. The median codon usage biases of the whole genome and coding sequences of ribosomal proteins (*n *=* *1,186; median number of ribosomal proteins per genome, 55; standard deviation, 3.6) were integrated in the network to compare the links between codons and tRNAs outside the methane oxidation module (details on the tRNA pool of each genome are given in Fig. S4e). The resulting networks (Fig. 3b; see Fig. S5a for networks for all amino acids) did not support the hypothesis that the codons favored by each operon provided differential access to tRNA isoacceptors. We also analyzed how access to different tRNA isoacceptors changes with variations in individual codon preferences of coding sequences (Fig. 3c). We found that the different codon usage biases do not provide the operons with access to significantly different fractions of the tRNA pool (chi-square test for equality of proportions, *P = *0.87). Taken together, these results suggest that the codon usage bias of type Ia *pmoCAB* coding sequences does not provide a fitness advantage either in translation efficiency or in differential access to different tRNA isoacceptors compared to those of the *mxaF* and *xoxF* coding sequences. Despite the differences in codon usage between operons from the methane metabolic module, simultaneous translation of these operons would still require competition for the same tRNA isoacceptors.

### Type Ia *pmoCAB* coding sequences optimize translation accuracy.

We hypothesized that the divergent codon bias of *pmoCAB* coding sequences might be a strategy to optimize translation accuracy. This strategy prevents the incorporation of incorrect amino acids in the nascent protein (28, 29) by reducing the binding probability of near-cognate tRNA isoacceptors (i.e., tRNAs that differ by 1 base from the correct anticodon) for the ribosomal A site (30). Such an optimization for accuracy can be achieved at the coding sequence level through specialization of the codon-anticodon interaction in which an amino acid is encoded by a frequent and optimal codon that is recognized by an abundant cognate tRNA (31). We hypothesized that if translation accuracy is a selective pressure acting on a coding sequence, the positive selection for an optimal codon (or the purifying selection acting on nonoptimal codons) should increase as that codon’s amino acid is increasingly used in the encoded protein.

To test this, we first calculated the amino acid usage of each protein (Fig. S5b) and mapped the RSCU with the highest median per amino acid (Fig. S4c) to the median frequency of amino acid usage. Finally, we used a linear regression to analyze the relationship between the codon preference in coding sequences and the amino acid usage frequency of their proteins. Consistently with our translational accuracy hypothesis, we found that among all methanotrophs and all coding sequences of the methane oxidation metabolic module, only the type Ia *pmoCAB* coding sequences showed a significant linear relationship (*P < *0.01) (Fig. 3d and Fig. S5c). This is especially notable for the two amino acids with the highest codon degeneracy (i.e., the 6-fold Leu and Arg).

### Minimizing protein synthesis cost through favoring of prebiotic amino acids.

The use of the CAI, ENC, RSCU, and tAI enabled us to investigate the role of synonymous codons in optimization for translation efficiency and accuracy. However, as these measures account for amino acid usage biases, optimization strategies at the protein sequence level can be masked. We hypothesized that if translation optimization at the protein sequence level is achieved through reducing the synthesis cost of proteins (32, 33), there should be an increased usage of the prebiotic (less expensive) amino acids (34) in the type Ia pMMO sequence. To test this, we first visually inspected the occurrence of prebiotic and modern amino acids in the linear regression between the RSCU and amino acid usage (Fig. 3d) and then quantitatively compared the median compositions of less expensive and expensive amino acids in each protein (Fig. 3e). We observed that the usage of prebiotic amino acids is significantly higher than that of modern (expensive) amino acids in pMMO, sMMO, and Xox and that Mxa showed a nonsignificant difference (*t* test, 99% confidence level) (Fig. 3e).

### The codon usage of *pmoCAB* coding sequences results in an effective resource allocation strategy to synthesize highly demanded transcripts.

While the codon usage and nucleotide composition biases of coding sequences in the type Ia methane oxidation module do not seem to provide an advantage in translation efficiency, it is possible that they provide an efficiency advantage at the level of transcription. Previous reports on methanotrophic species (35–37) and communities (38–40) have shown that *pmoCAB* coding sequences are consistently highly expressed regardless of the experimental conditions, and the demand on cellular resources to transcribe *pmoCAB* coding sequences may be significant. To test whether the biases confer increased transcriptional efficiency, the transcriptomic data sets of three different type Ia methanotrophs obtained under different experimental conditions were examined. For illustration purposes, the results of Methylobacter tundripaludum 31/32 grown with methane as the sole carbon source and supplemented with lanthanum (37) are described here. Detailed results with the other methanotrophs and experimental conditions can be found in Fig. S6.

10.1128/mSystems.00342-19.6 FIG S6 Strategies for optimal transcription in Methylobacter tundripaludum 31/32, Methylomicrobium alcaliphilum 20Z, and Methylomicrobium buryatense 5G. (a) Frequency of purine and pyrimidine contents in all coding sequences. (b) Normalized mRNA abundances and the pyrimidine contents of the corresponding coding sequences of methanotrophs growing under different conditions. (c) Mean number of pyrimidines per base pair of the *pmoCAB* coding sequences and the mean number of pyrimidines per base pair of 1,000 random samples of three coding sequences (*n *=* *3). (d, e, f) Effects on the total ribonucleotide composition due to removal of a set of transcribed coding sequences from the transcriptome (T). (g, h) Elemental compositions of transcribed coding sequences. The main panel shows the number of element atoms per codon in each transcribed coding sequence, with the dashed lines representing the mean of all transcribed coding sequences in the genome. The top and right panels show the mean number of element atoms per codon of the transcribed *pmoCAB* coding sequences and the mean number of element atoms per codon of 1,000 random samples of three transcribed coding sequences. (i) Correlation between elemental composition and mRNA abundance. The Pearson correlation coefficient with 95% confidence levels is depicted in each panel. Download FIG S6, PDF file, 2.1 MB. Copyright © 2019 Villada et al. 2019 Villada et al. This content is distributed under the terms of the Creative Commons Attribution 4.0 International license.

To test the transcription optimization hypothesis, we first analyzed the purine and pyrimidine contents of coding sequences in each genome and found that purine-derived residues occurred more frequently (52%) than pyrimidine nucleotides (Fig. S6a). We postulated that, if *pmoCAB* coding sequences are optimized for efficient transcription, they should have a higher pyrimidine than purine content, which reduces the mRNA synthesis cost, as pyrimidines have fewer carbon and nitrogen atoms per molecule (Fig. S6a). We found that *pmoCAB* were the most expressed transcripts and that they have a higher pyrimidine than purine content (Fig. S6b), contrasting with the *xoxF* and *mxaFI* coding sequences (Fig. S6b) and the genome-scale frequency (Fig. S6a). We calculated the mean pyrimidine content of 1,000 random combinations of three coding sequences (Fig. S6c) and found that <3.5% of the combinations had pyrimidine contents higher than those of the *pmoCAB* coding sequences.

To understand the effect of *pmoCAB* nucleotide frequency bias on cellular ribonucleotide composition, we analyzed the effect on the total ribonucleotide composition if we removed all the transcripts of an individual operon from the transcriptome. We calculated the total ribonucleotide composition by summing the ribonucleotide compositions of all transcribed coding sequences in the transcriptome. We found that removing *mxaFI* and *xoxF* transcripts had a negligible effect on the total ribonucleotide composition (Fig. S6d to f). However, removing *pmoCAB* transcripts caused a shift of ∼1% in the purine/pyrimidine balance, a shift of >2% in the GC content, and a shift of ∼2% in uracil content (Fig. S6d to f).

**FIG 4:**
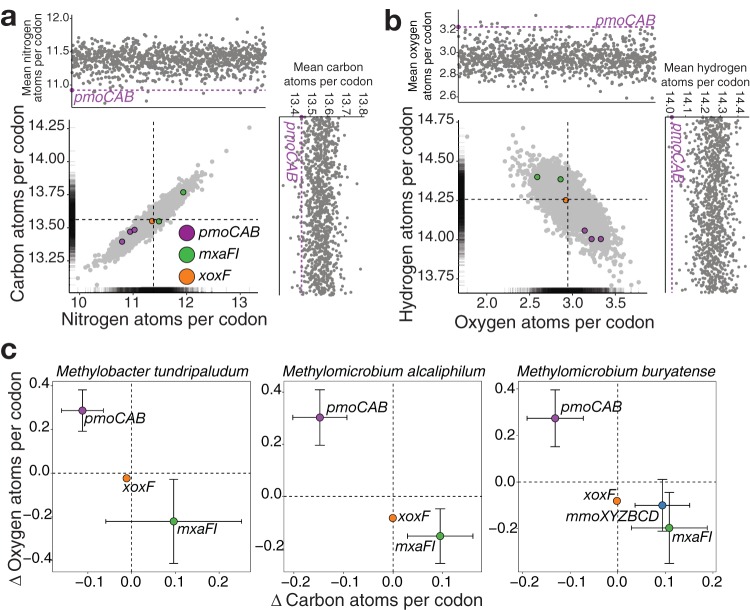
Strategies for optimal transcription in type Ia methanotrophs. (a, b) Elemental compositions of transcribed coding sequences in *M. tundripaludum* 31/32. The main panel shows the number of element atoms per codon in each transcribed coding sequence, with the dashed lines representing the mean of all transcribed coding sequences in the genome. The top and right panels show the mean number of element atoms per codon of the *pmoCAB* transcribed coding sequences and the mean number of element atoms per codon of 1,000 random combinations of three coding sequences. (c) Summary of the carbon and oxygen atom demands per codon of transcribed coding sequences. The difference (Δ) was calculated using the genome-scale mean demand as the reference (dashed line). *M. buryatense* 5G possesses genes encoding both the pMMO and the sMMO. An extended analysis for each methanotroph is shown in Fig. S6.

If the nucleotide composition bias of *pmoCAB* coding sequences is an adaptation to reduce transcript synthesis cost, it is expected that the atomic element investment per codon would be reduced in transcribed *pmoCAB* coding sequences compared to those of others in the methane oxidation metabolic module and to that of the whole-genome average. To evaluate this, we calculated the atomic element compositions of each transcribed coding sequence in the metabolic module and of all transcribed coding sequences in the genome based on their adenine, guanine, cytosine, and uracil compositions. We found that *pmoCAB* transcripts have fewer carbon, hydrogen, and nitrogen atoms per codon than other transcripts from the methane metabolic module and the genome-scale mean (Fig. 4a and b). However, although the codon usage of *pmoCAB* transcripts requires reduced investment of carbon, nitrogen, and hydrogen, this comes at the expense of increased oxygen demand, with the oxygen demand exceeding the genome-scale mean (Fig. 4b). We selected 1,000 random combinations of three transcribed coding sequences from the whole genome, calculated the per-codon element compositions, and compared them to the demand of the transcribed *pmoCAB* coding sequences (Fig. 4a and b). We found that <3% of the combinations of transcribed coding sequences yielded a mean element composition below that of the transcribed *pmoCAB* coding sequences for nitrogen, carbon, and hydrogen or above that for oxygen. This pattern was even more pronounced for Methylomicrobium alcaliphilum 20Z (<0.4%) (Fig. S6g and h) and Methylomicrobium buryatense 5G (<1.5%) (Fig. S6g and h). Next, we calculated the distance (Δ) between the mean carbon and oxygen atom counts per codon for methane oxidation metabolic module transcripts and all transcripts from each genome. We found that the *pmoCAB* transcripts had low carbon and high oxygen compositions per codon (Fig. 4c). Finally, we assessed whether the elemental composition of the *pmoCAB* operon is a result of a genome-wide trend to reduce the number of atoms per codon as a function of mRNA abundance. Rejecting this hypothesis, we found only weak correlations between elemental content and mRNA abundance (Fig. S6i). Together, these results suggest an optimization strategy unique to type Ia *pmoCAB* coding sequences whereby highly demanded transcripts use fewer carbon, nitrogen, and hydrogen atoms at the expense of more oxygen atoms per codon. Given the high oxygen requirement for the synthesis of *pmoCAB* transcripts, we hypothesized that type Ia genomes may harbor a genetic regulatory mechanism adjusting *pmoCAB* transcription in response to oxygen availability. In support of this, we identified proteins containing the PAS domain which can potentially act as oxygen sensors (41), located near the *pmoCAB* coding sequences in the type Ia genomes of the genera *Methylosarcina*, *Methylomonas*, *Methylomicrobium*, and *Methylomarinum* (about 10 coding sequences upstream) and the species Methylobacter tundripaludum 31/32 (5 coding sequences downstream) (Fig. S7).

10.1128/mSystems.00342-19.7 FIG S7 Location of the PAS domain proteins relative to the *pmoCAB* coding sequences in different type Ia methanotrophs. A negative value in the *x* axis indicates the number of coding sequences upstream of the *pmoCAB* coding sequences, and a positive value indicates downstream coding sequences. Download FIG S7, PDF file, 0.1 MB. Copyright © 2019 Villada et al. 2019 Villada et al. This content is distributed under the terms of the Creative Commons Attribution 4.0 International license.

## DISCUSSION

This study provides genomic evidence that the *pmoCAB* coding sequences of type Ia methanotrophs possess a unique adaptive trait manifesting as a strong nucleotide bias (Fig. 1) that fine-tunes codon usage (Fig. 2) and can optimize methane oxidation through maximizing translation efficiency and accuracy (Fig. 3), while minimizing protein (Fig. 3) and transcript (Fig. 4) synthesis costs. The discovery of this unique coding sequence was enabled by meta-analysis of a large number of isolate genomes and MAGs from diverse taxonomies and geographies (Fig. S1). This finding illustrates a sophisticated adaptive linkage between molecular genotype and phenotype for methane oxidation. Given the high metabolic modularity of methanotrophy (10, 42), the methods presented here can be applied to probe the molecular strategies encoded in other functional modules (e.g., copper uptake; *mbnABCM*, *corAB*, and *copCD*) that are of particular relevance to our understanding of methanotrophs.

From an environmental point of view, this adaptive trait enables type Ia methanotrophs to be competitive and efficient in oxidizing methane, which is a gas with limited solubility and mass transfer in liquid (43). Past reports on the molecular optimization of genetic sequences have shown that transcribed regions of genes can have fine-tuned nucleotide compositions that reduce their biosynthetic cost (24). For example, DNA coding sequences are found to reduce the number of nitrogen atoms per codon when nitrogen is scarce in the environment (44), and a large proportion of bacterial genomes present a dual-optimization strategy to reduce per-codon nitrogen demand while increasing translation efficiency (45). Extending these findings, we show that type Ia *pmoCAB* coding sequences possess an adaptation that minimizes not only nitrogen but also carbon and hydrogen per transcribed codon, while reducing the metabolic burden of erroneous biosynthesis (30) through optimal translation efficiency and accuracy. This optimization strategy may increase the fitness of type Ia methanotrophs in the competition for methane and might contribute to the prevalence of type Ia methanotrophs across diverse habitats with various environmental conditions (7). However, given that the number of sequenced genomes available for other types of methanotrophs is limited, we might find alternative optimization strategies at the molecular level with future sequencing projects. Nonetheless, the transcript optimization strategy found in type Ia *pmoCAB* coding sequences results in an increased demand for oxygen atoms per codon. The high demand for oxygen to synthesize *pmoCAB* transcripts may be part of a mechanism to modulate cellular metabolism based on oxygen availability in the environment and to tightly regulate the first enzymatic step of the methane oxidation metabolic module at the transcriptional level. This is supported by the presence of genes encoding the PAS domain proteins, potentially acting as oxygen sensors (41), flanking the *pmoCAB* coding sequences in the genomes of several type Ia methanotrophs (Fig. S7).

**FIG 5:**
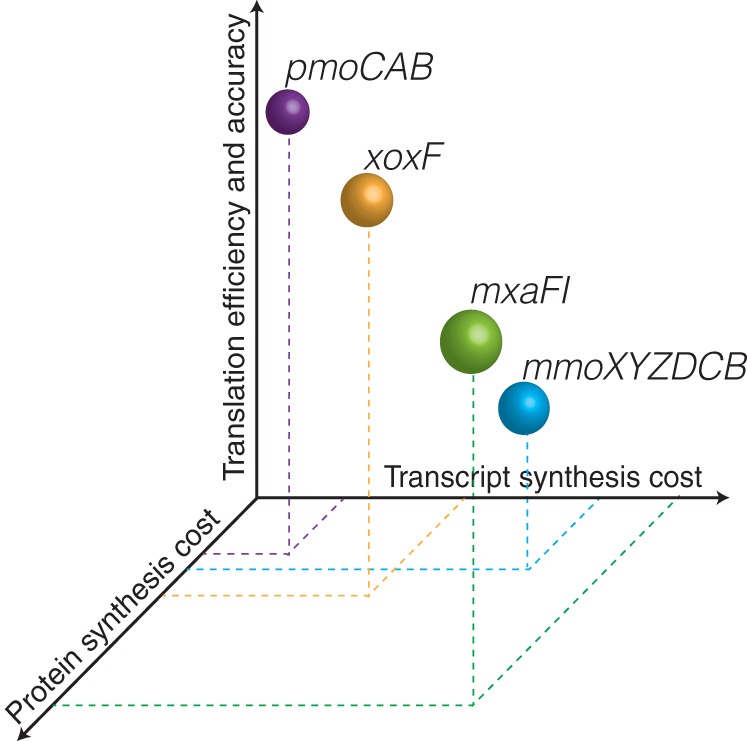
Three dimensions of molecular optimization encoded in the coding sequences of the methane oxidation metabolic module of type Ia methanotrophs.

In the second enzymatic step of the methane oxidation metabolic module, methanol is oxidized to formaldehyde by the methanol dehydrogenase encoded by either the *mxaFI* or *xoxF* gene. We show here that the type Ia *pmoCAB*, *mxaFI*, and *xoxF* coding sequences have similar translation efficiencies (Fig. 3a), similar levels of access to tRNA isoacceptors (Fig. 3b and c), and similar levels of codon bias (Fig. 2b); however, their preferences for individual codons (Fig. S2a) and translation accuracy (Fig. 3d) differ. Considering only the genes for the second enzymatic step, the difference in codon usage between the *mxaFI* and *xoxF* coding sequences reduces the *xoxF* transcripts’ demand for carbon, nitrogen, and hydrogen atoms per codon (Fig. 4). We also show that protein synthesis has been optimized through the frequent incorporation of prebiotic (inexpensive) amino acids in the pMMO and Xox protein sequences, though not in Mxa (Fig. 3e). Together, these results indicate that although the *mxaFI* and *xoxF* coding sequences have similar translation efficiencies, the *xoxF* coding sequence reduces transcript and protein synthesis costs (Fig. 5), suggesting that Xox has evolved to be the predominantly expressed methanol dehydrogenase. Overall, the results in this study show that the pMMO-Xox configuration of the methane oxidation module should be the most efficient molecular strategy to catalyze the sequential oxidation of methane and methanol (Fig. 5). This finding complements the results of recent investigations of isolates (46) and communities (47) by providing a molecular basis to explain the prevalent expression of *pmoCAB* and *xoxF* coding sequences. Future investigations focusing on the implications of the *pmoCAB* and *xoxF* coding sequences in the context of ecogenomics and ecophysiology will be required.

Genes encoding pMMO have been scrutinized for nucleotide composition bias (48, 49), and it has been proposed that they have evolved independently following an ancient speciation event (50). An earlier work indicated that methanotrophy in *Alphaproteobacteria* was a result of horizontal gene transfer of MMOs (11). In fact, recent evidence supports the horizontal transfer of MMO genes to methanotrophs in *Proteobacteria* (12–14). In the context of the literature, the most parsimonious explanation for our observations is that *pmoCAB* genes were acquired through an ancient horizontal gene transfer event and subsequently subjected to strong selective pressure that restricts gene amelioration (51) to the nucleotide composition of the type Ia genomes, which preserves a foreign codon usage bias that optimizes the transcriptional and translational machinery (52).

Fundamentally, our results emphasize that the type Ia *pmoCAB* coding sequences have been shaped by natural selection and are not the result of random genetic drift even at the level of synonymous codons. The ecological role of methanotrophs as the primary producers in complex communities (5, 6) can exert strong evolutionary pressures on pMMO. Efficient conversion of methane to methanol not only serves as the first enzymatic step for methanotrophs but also provides methanol as the carbon source for methylotrophic bacteria (37). The availability of methanol can also support methane oxidation in methanotrophs that have a low affinity for methane when methane concentration is at atmospheric levels (53). Consequently, the unique adaptations of the type Ia *pmoCAB* coding sequences are likely the result of molecular evolution at the organismal, community, and perhaps even planetary scale (54).

## MATERIALS AND METHODS

Detailed description of the methods used in this study is available in Text S1 in the supplemental material.

10.1128/mSystems.00342-19.10 TEXT S1 Supplemental methods. Download Text S1, PDF file, 0.1 MB. Copyright © 2019 Villada et al. 2019 Villada et al. This content is distributed under the terms of the Creative Commons Attribution 4.0 International license.

### Methanotroph genomes.

Of the 59 methanotroph genomes analyzed in this study, 47 are isolates and 12 are metagenome-assembled genomes (MAGs) (see Table S1 for details). This collection included most of the isolate genomes and relevant methanotrophic metagenomic data sets available in the public domain as of October 2017. Genome annotations were compiled from the JGI/IMG (55), NCBI/RefSeq (56), and KEGG/BlastKOALA (57) databases. The phylogenies of the genomes were assigned by PhyloPhlAn (58).

### Metagenomic analysis pipeline.

Briefly, raw reads of publicly available metagenomic data sets were obtained from the NCBI. Quality control of raw reads was performed with illumina-utils (59). Contigs were coassembled with MEGAHIT (60), and binning was performed with MaxBin (61). The quality of MAGs was assessed with CheckM (62) and MAGs were categorized according to the quality standards defined in the article “Minimum Information about a Single Amplified Genome (MISAG) and a Metagenome-Assembled Genome (MIMAG) of Bacteria and Archaea” (63). Annotation of MAGs was conducted with Prokka (64) and eggNOG-mapper (65).

### Sequence analysis.

Nucleotide composition bias was analyzed with seqinR (66) in R. Codon usage metrics were formulated in R according to the equations described for the RSCU (67) and CAI (67). tAIs were calculated with codonR (68). ENCs (69) were calculated with chips in EMBOSS (70). The tRNA data sets required for the reproduction of tAI calculations can be found in Table S2. The network data set was generated using in-house scripts. The network matrix was imported and edited with Cytoscape (71). Whole-genome analyses of codon metrics were conducted as detailed elsewhere (72).

10.1128/mSystems.00342-19.9 TABLE S2 Detailed information of the tRNA pool of each genome. Download Table S2, XLSX file, 0.02 MB. Copyright © 2019 Villada et al. 2019 Villada et al. This content is distributed under the terms of the Creative Commons Attribution 4.0 International license.

### Transcriptomic analysis.

Normalized mRNA abundance data from RNA-Seq experiments were obtained from three publicly available independent studies conducted with the type Ia methanotrophs Methylomicrobium buryatense 5G (35), Methylomicrobium alcaliphilum 20Z (36), and Methylobacter tundripaludum 31/32 (37).

### Data availability.

Scripts required to reproduce all the results and figures can be obtained from https://github.com/PLeeLab/methane_oxidation_genetic_trait/.
