# Supplementary figures and images for: Genomic Evidence for Simultaneous Optimization of Transcription and Translation through Codon Variants in the pmoCAB Operon of Type Ia Methanotrophs
Source: mSystems. 2019 Jul 23;4(4):e00342-19. doi: 10.1128/mSystems.00342-19 (PMC6650546; doi:10.1128/mSystems.00342-19)

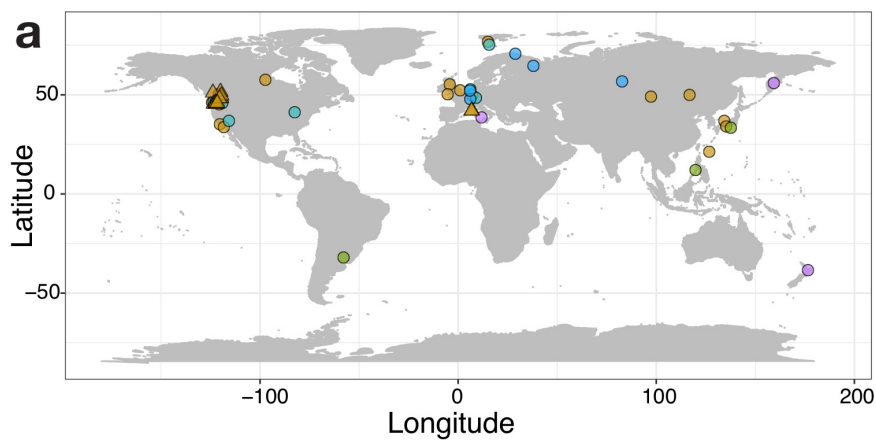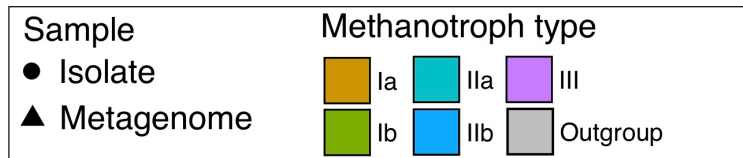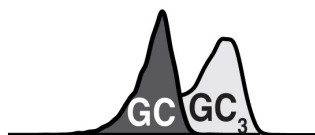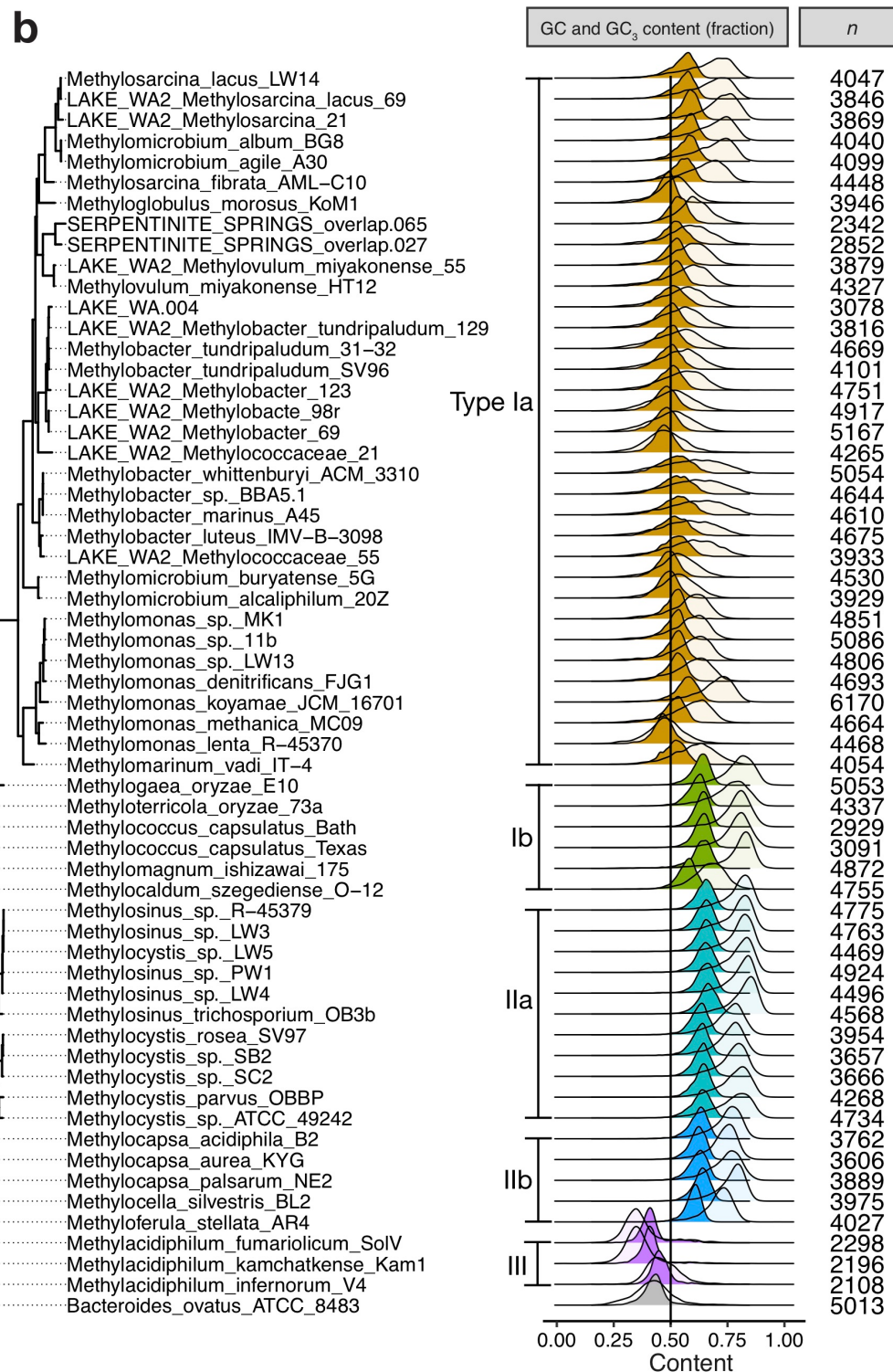

**C**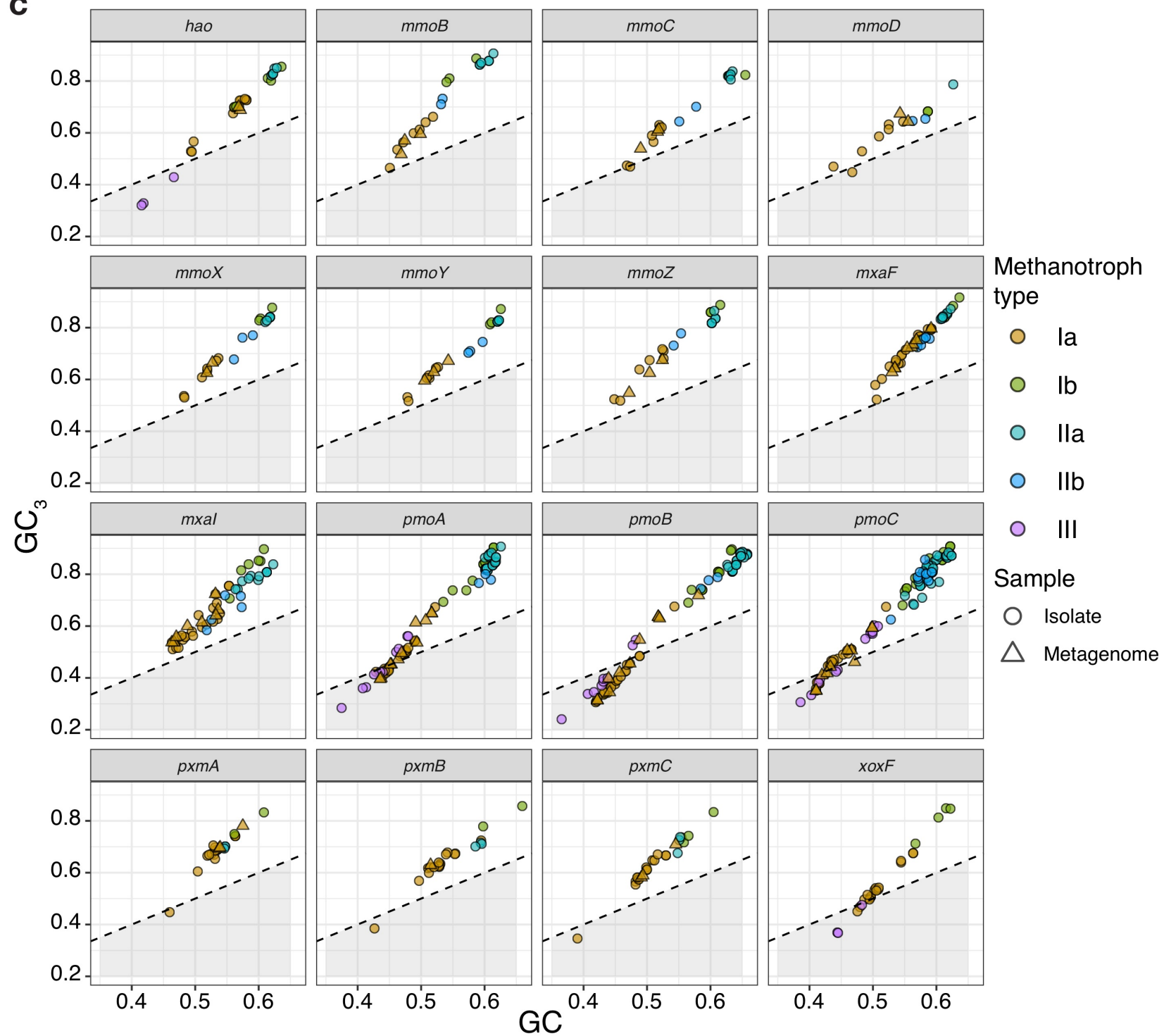

Supplement: FIG S1 [file mSystems.00342-19-sf001.pdf]

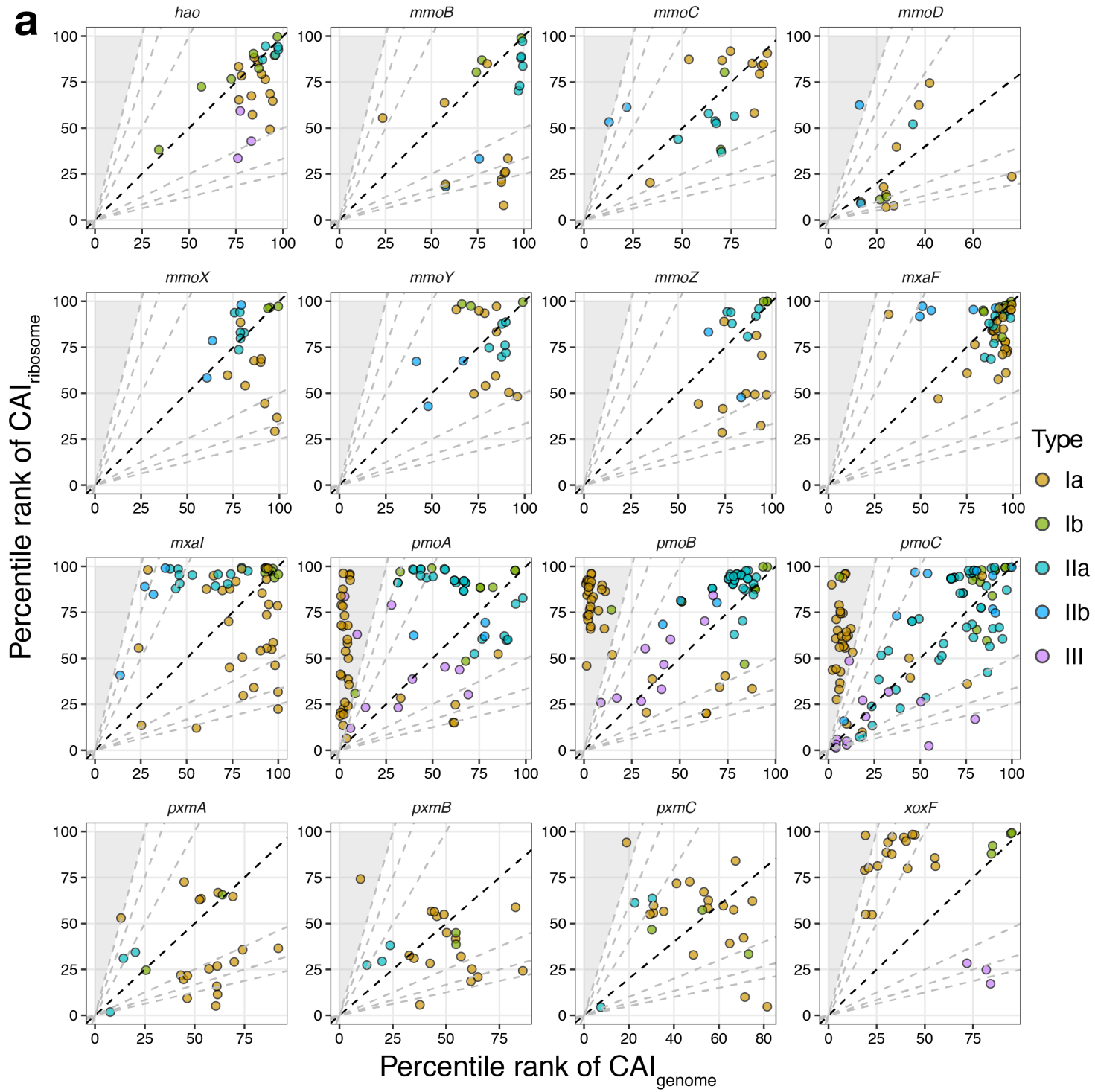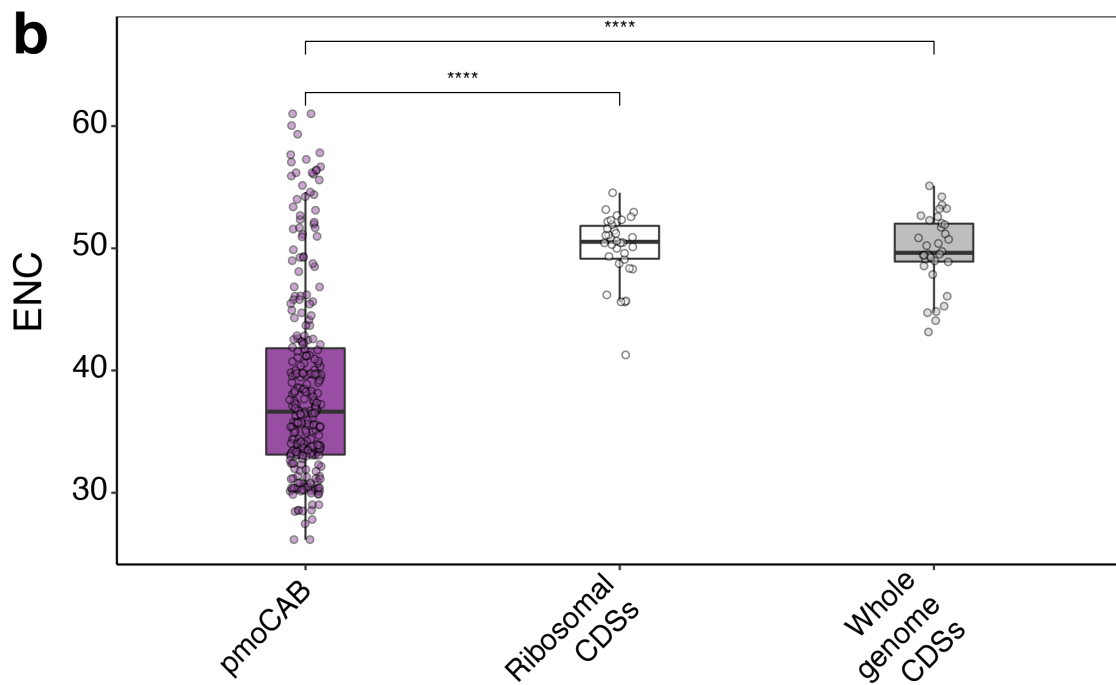

**C**

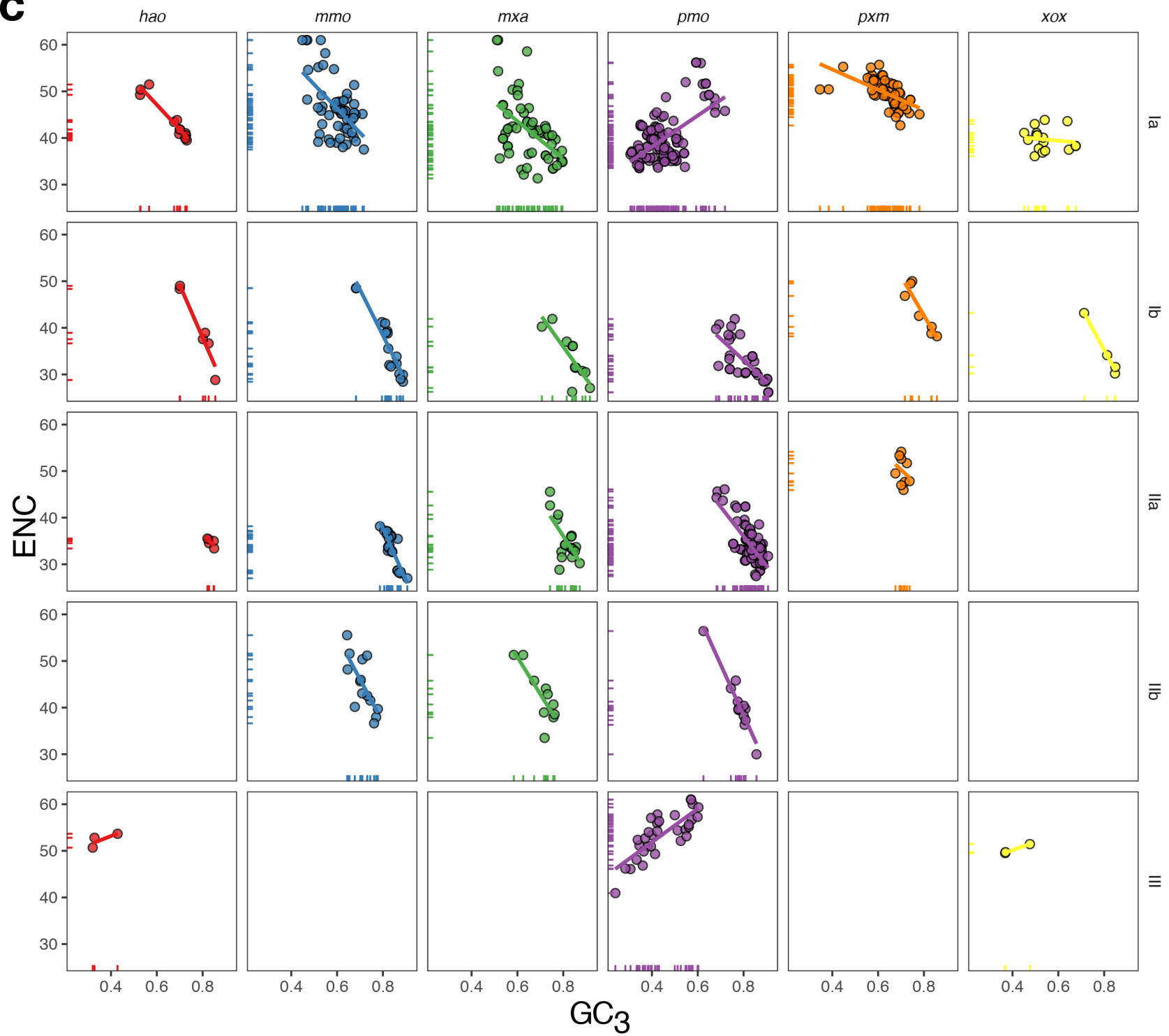

Supplement: FIG S3 [file mSystems.00342-19-sf003.pdf]

**a**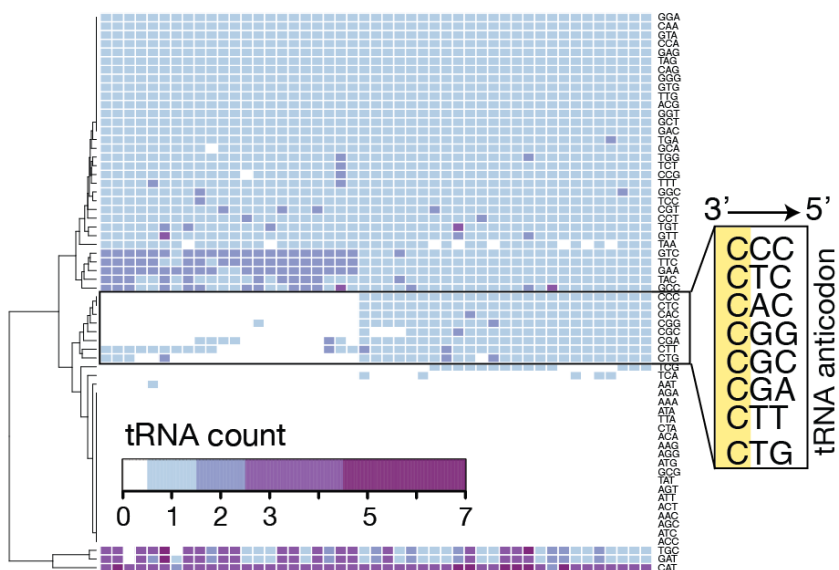**b**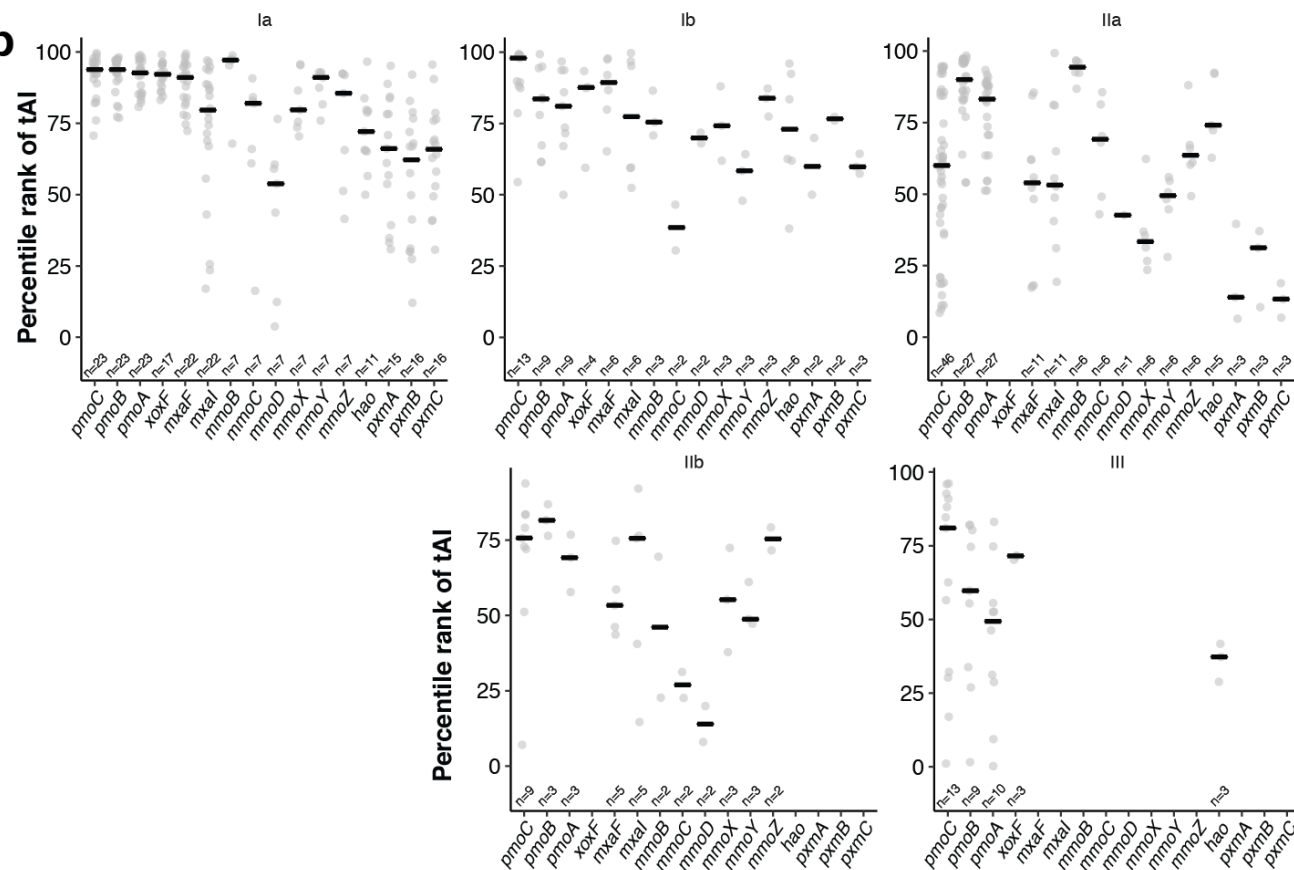**c***pmoCAB**mnoXYZDCB**mxaFI**soxF*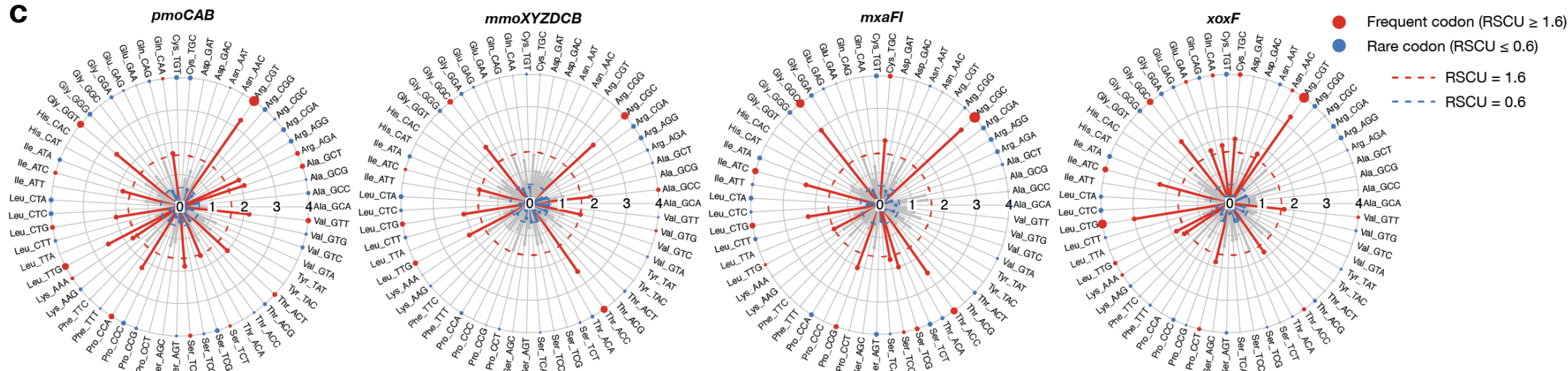

**d**

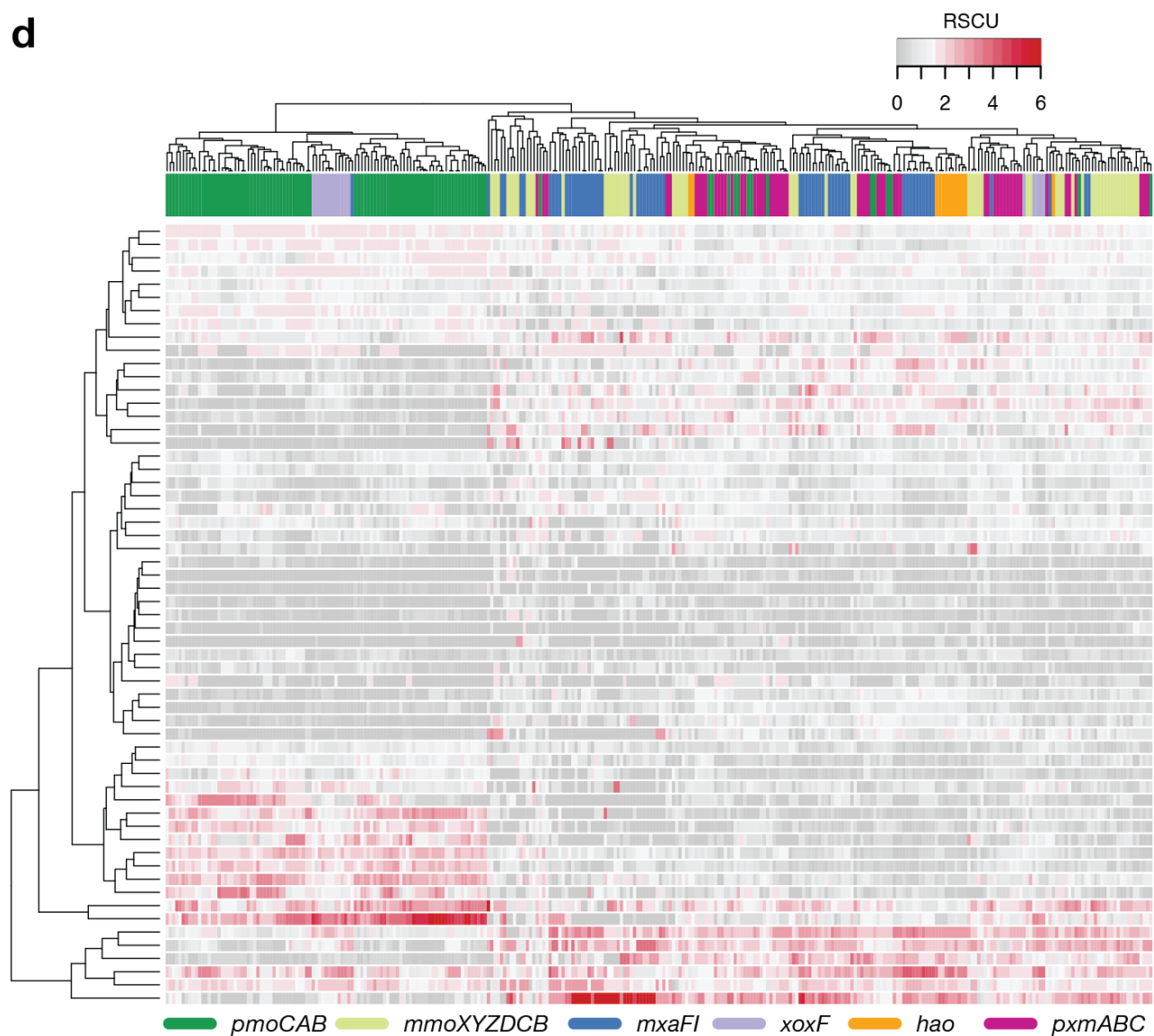

**e**

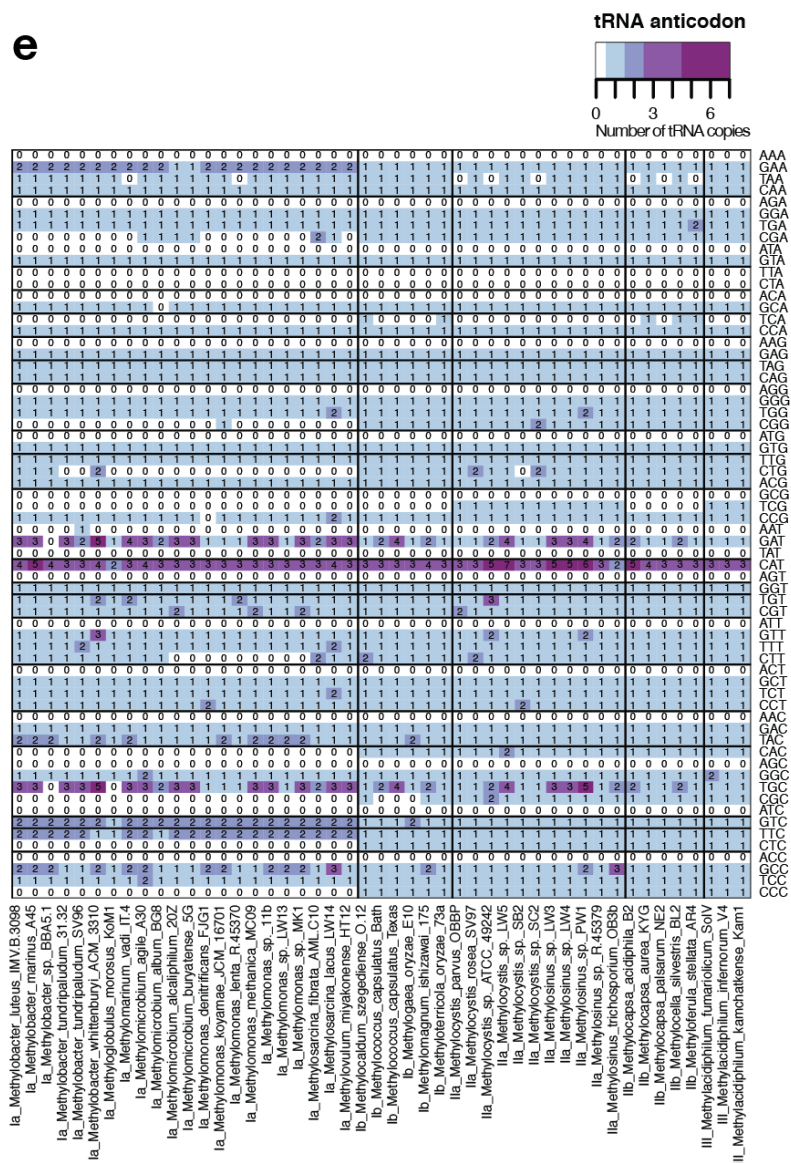

Supplement: FIG S4 [file mSystems.00342-19-sf004.pdf]

**a**

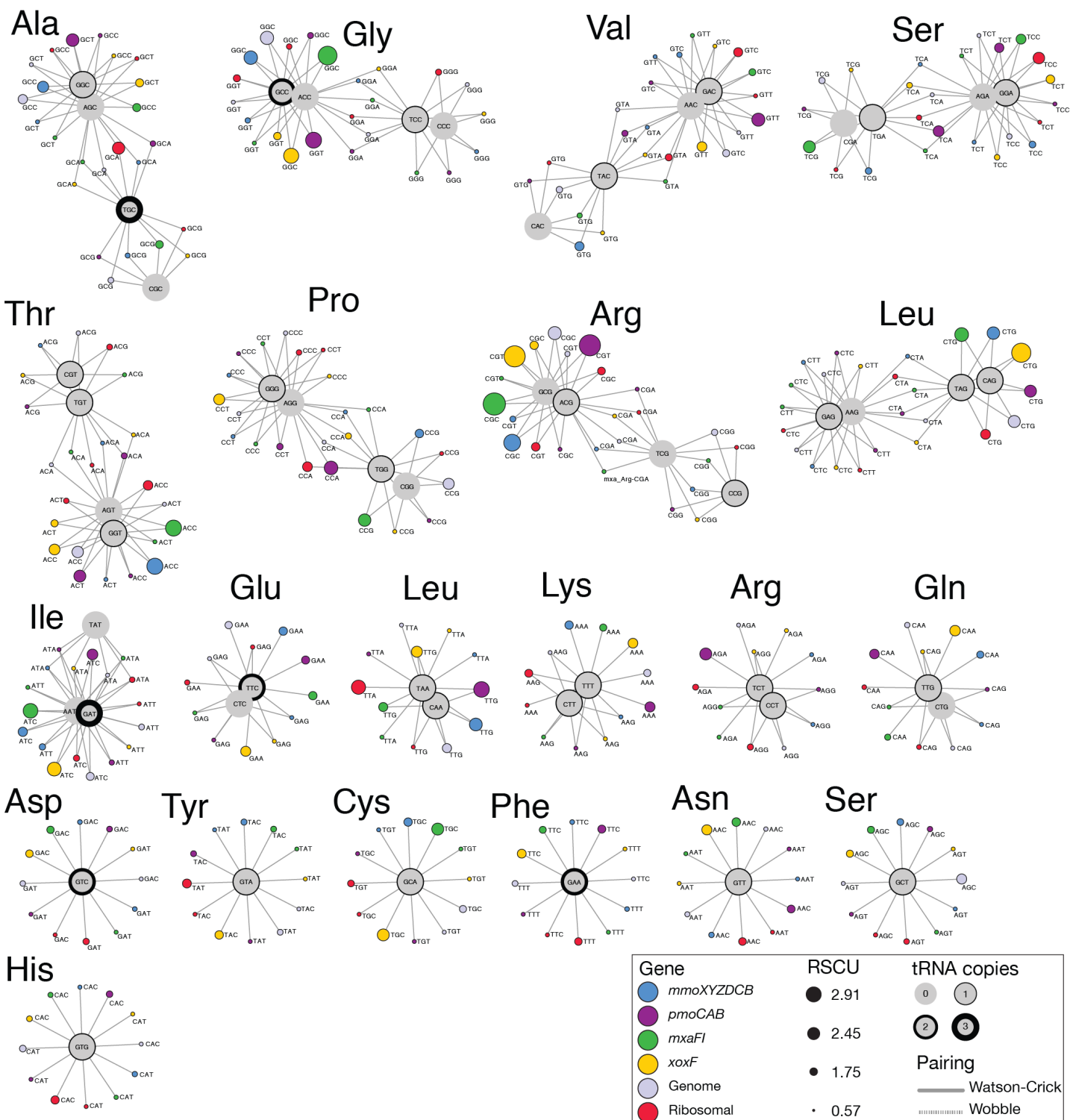

**b**

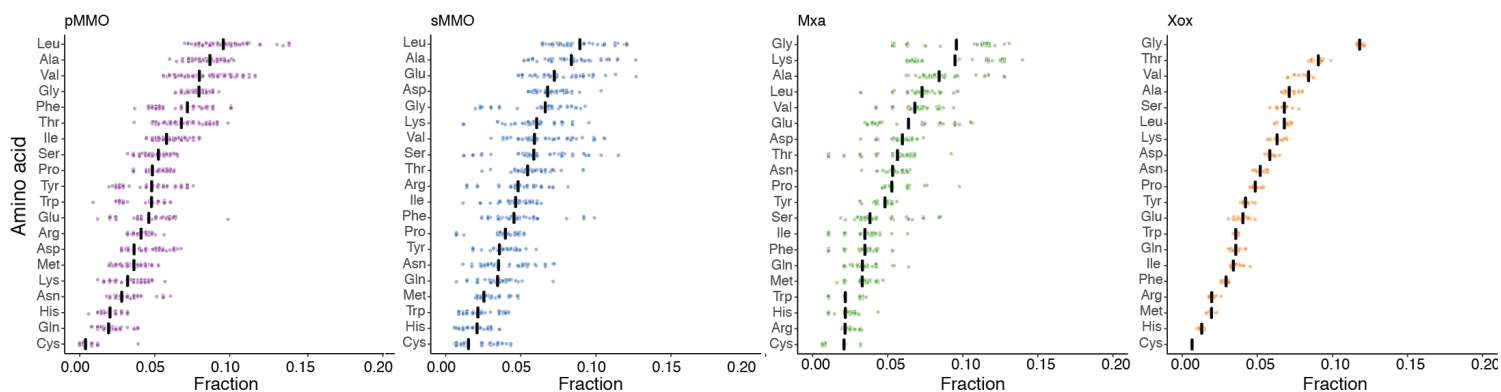

C

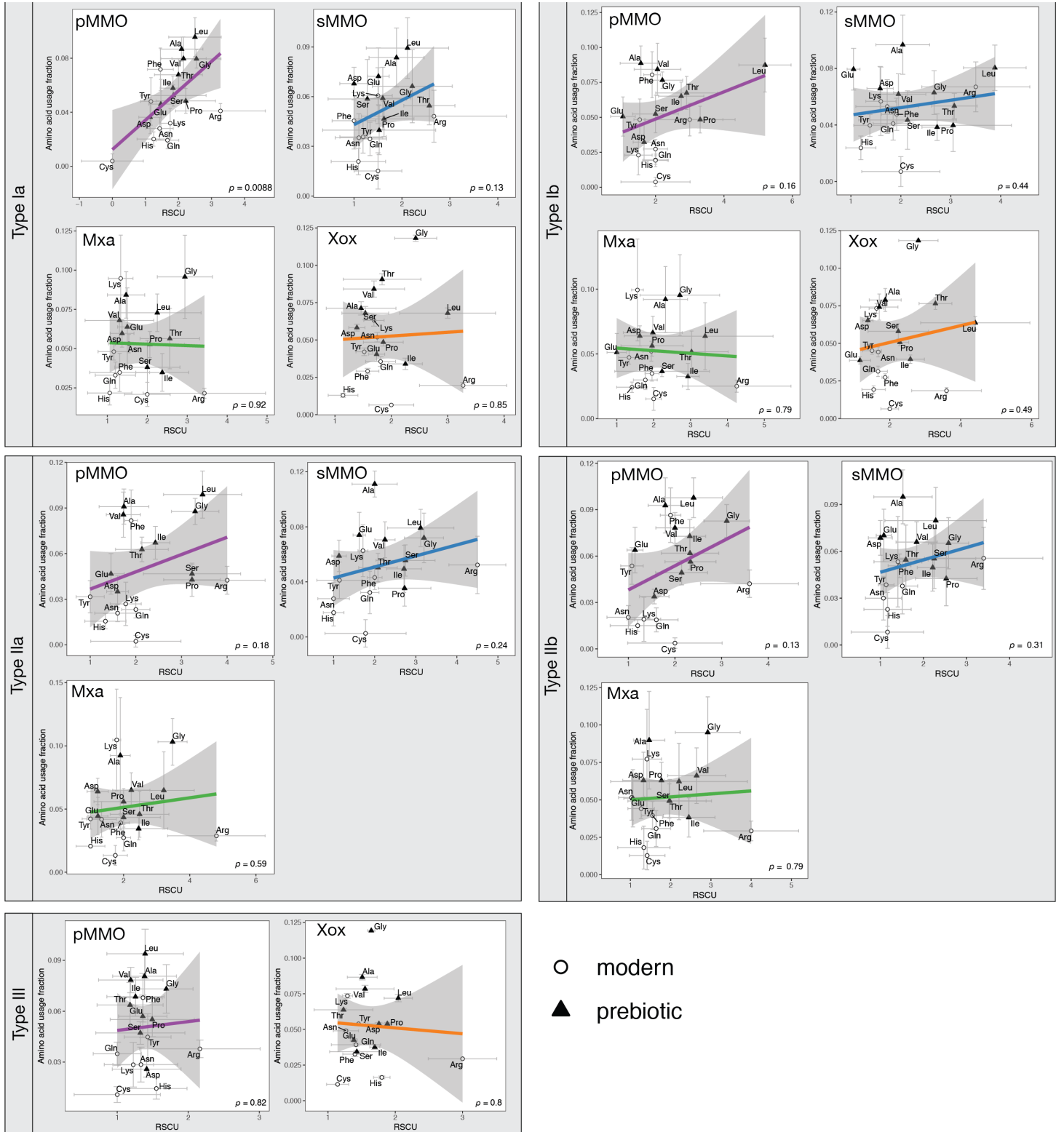

Supplement: FIG S5 [file mSystems.00342-19-sf005.pdf]

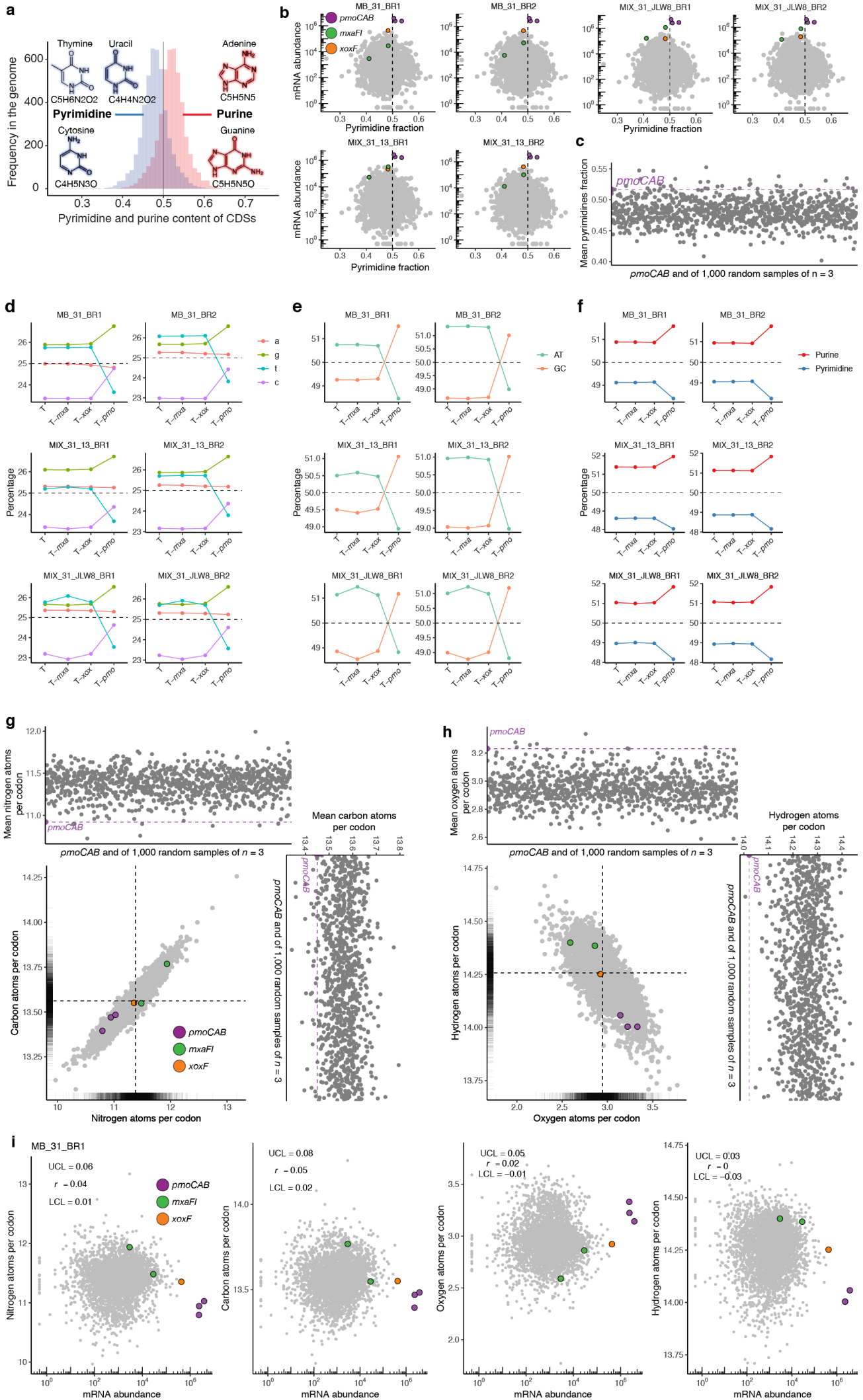

# Methylomicrobium alcaliphilum 20Z

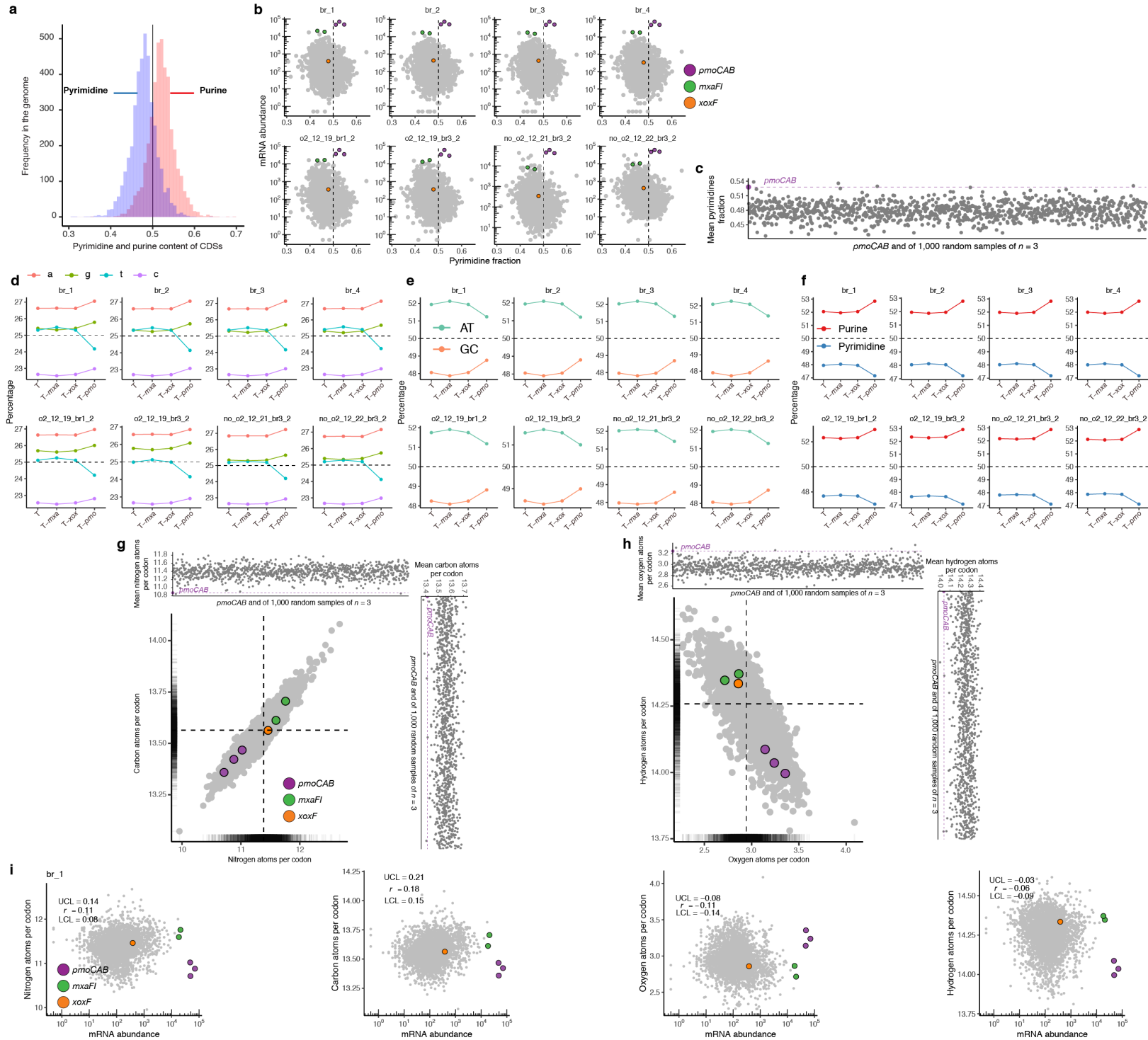

# Methylobacterium buryatense 5G

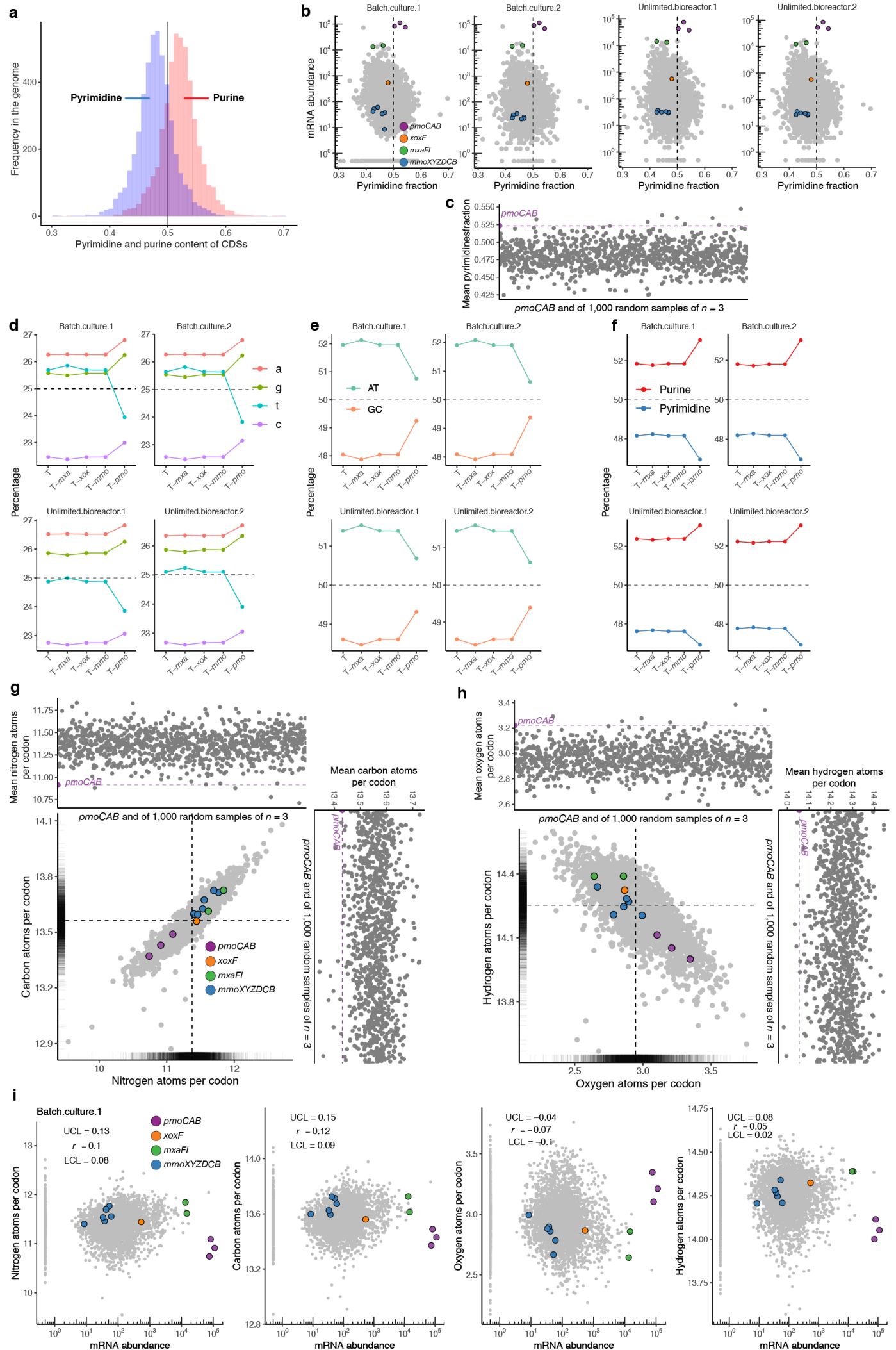

Supplement: FIG S6 [file mSystems.00342-19-sf006.pdf]

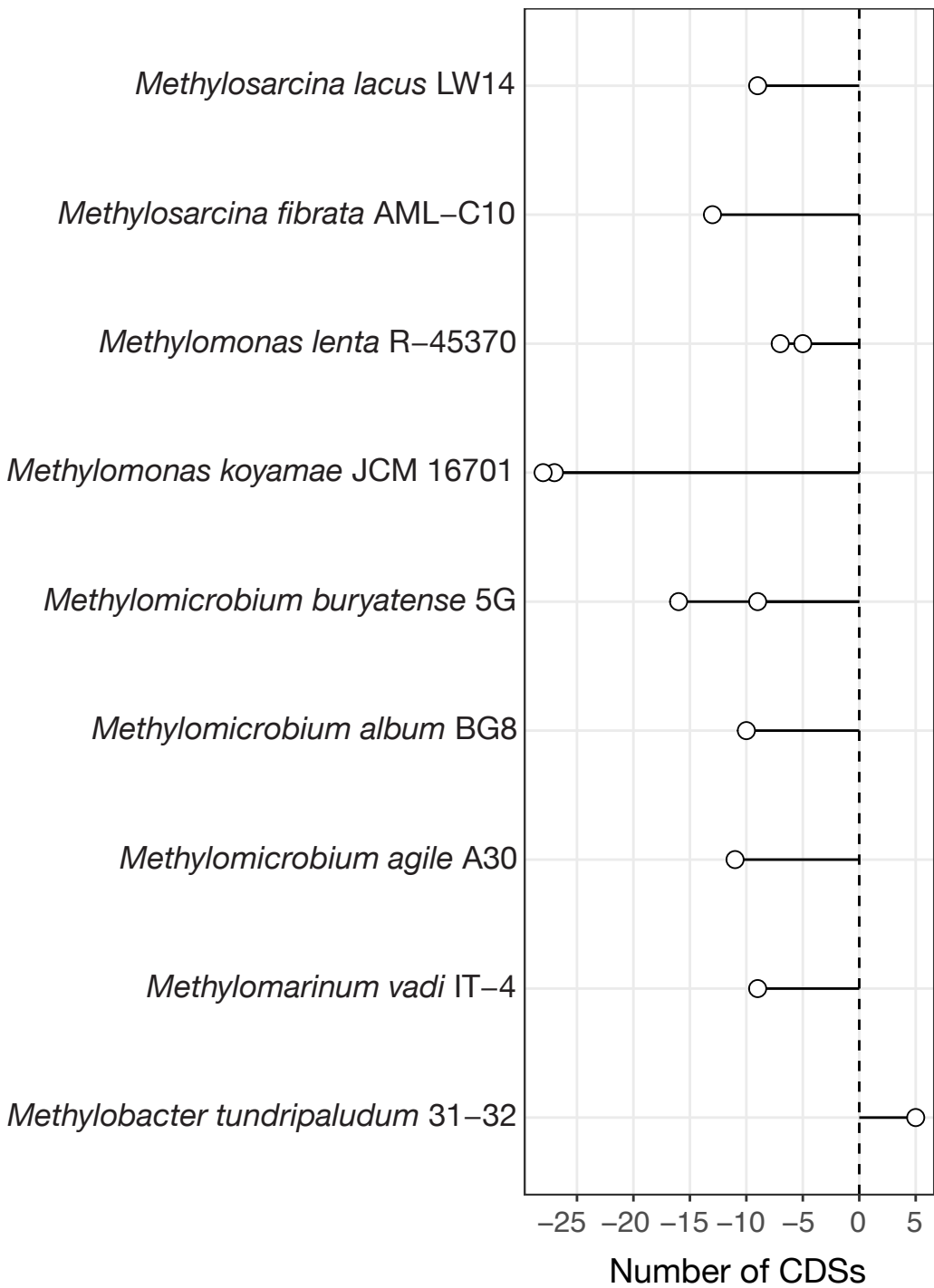

Supplement: FIG S7 [file mSystems.00342-19-sf007.pdf]
