# Supplementary material for: Genomic Evidence for Simultaneous Optimization of Transcription and Translation through Codon Variants in the pmoCAB Operon of Type Ia Methanotrophs
Source: mSystems. 2019 Jul 23;4(4):e00342-19. doi: 10.1128/mSystems.00342-19 (PMC6650546; doi:10.1128/mSystems.00342-19)
Supplement: FIG S2 [file mSystems.00342-19-sf002.pdf]

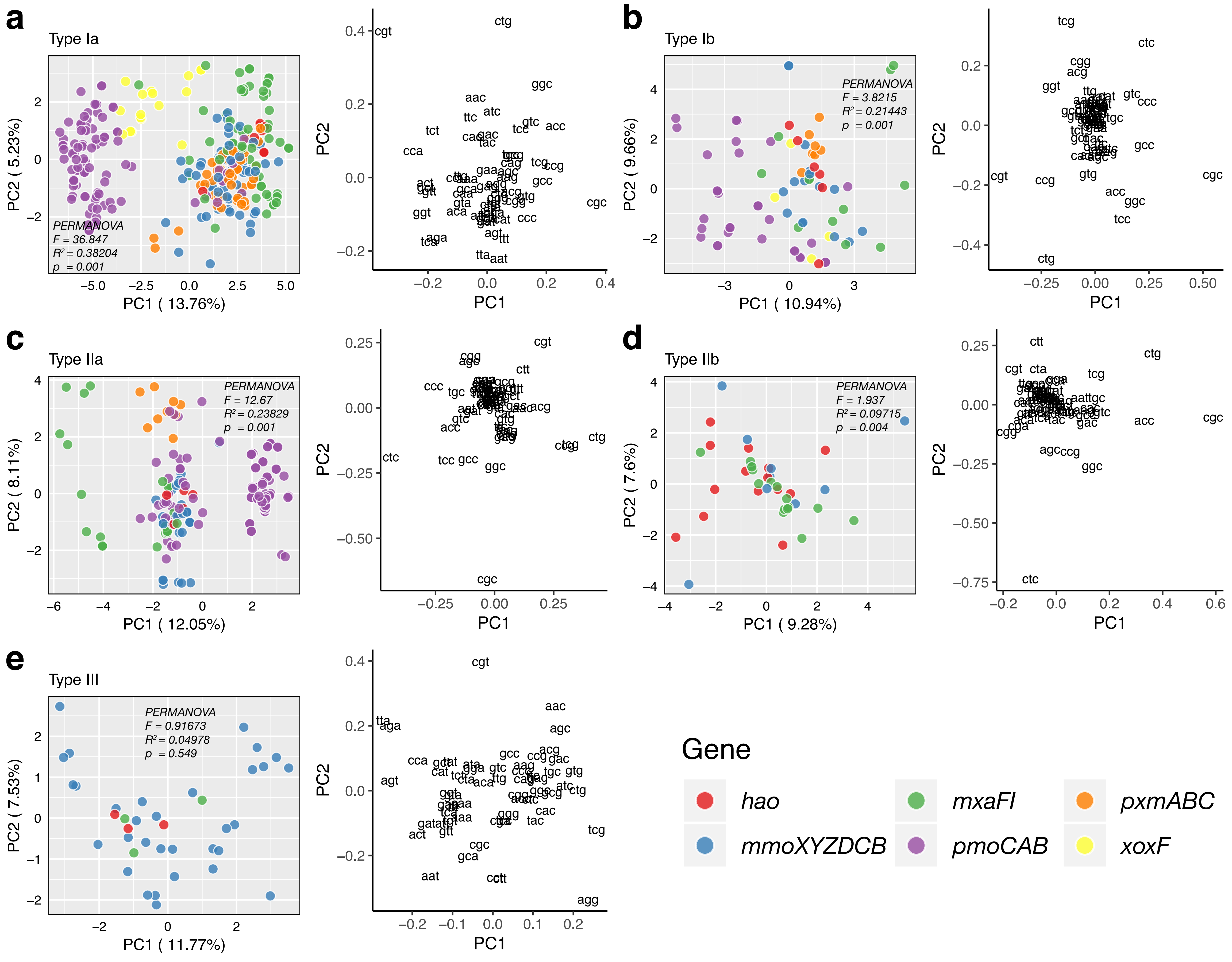
a
Type Ia
PERMANOVA
F = 36.847
R² = 0.38204
p = 0.001
PC1 (13.76%)
PC2 (5.23%)
PC1
PC2
b
Type Ib
PERMANOVA
F = 3.8215
R² = 0.21443
p = 0.001
PC1 (10.94%)
PC2 (9.66%)
c
Type IIa
PERMANOVA
F = 12.67
R² = 0.23829
p = 0.001
PC1 (12.05%)
PC2 (8.11%)
d
Type IIb
PERMANOVA
F = 1.937
R² = 0.09715
p = 0.004
PC1 (9.28%)
PC2 (7.6%)
e
Type III
PERMANOVA
F = 0.91673
R² = 0.04978
p = 0.549
PC1 (11.77%)
PC2 (7.53%)
Gene
hao
mxaFI
pxmABC
mmoXYZDCB
pmoCAB
xoxF
