# Supplementary material for: Genomic Evidence for Simultaneous Optimization of Transcription and Translation through Codon Variants in the pmoCAB Operon of Type Ia Methanotrophs
Source: mSystems. 2019 Jul 23;4(4):e00342-19. doi: 10.1128/mSystems.00342-19 (PMC6650546; doi:10.1128/mSystems.00342-19)
Supplement: TEXT S1 [file mSystems.00342-19-s0001.pdf]

Supplemental Material

Genomic evidence for simultaneous optimization of transcription and translation through codon variants in the *pmoCAB* operon of type Ia methanotrophs

Juan C. Villada, Maria F. Duran, and Patrick K. H. Lee[#]

School of Energy and Environment, City University of Hong Kong, Kowloon, Hong Kong SAR, China

Correspondence: [#]B5423, Yeung Kin Man Academic Building, School of Energy and Environment, City University of Hong Kong, Tat Chee Avenue, Kowloon, Hong Kong SAR, China; E-mail: patrick.kh.lee@cityu.edu.hk; Tel: (852) 3442-4625; Fax: (852) 3442-0688.

Supplemental Methods

Computational packages and scripts. *R* [1], *RStudio* [2], and *ggplot2* [3] were used to produce all the analyses and figures presented in this study unless otherwise indicated. All the scripts used in this work are available at GitHub (https://github.com/PLeeLab/methane_oxidation_genetic_trait).

Metagenomics analysis pipeline. Our analysis was applied to publicly available metagenomic data from five potentially methanotrophic environments: 1) Lake Washington, USA [4]; 2) Serpentinite Springs of the Voltri Massif, Italy [5]; 3) Movile Cave in Mangalia, Romania [6]; 4) Santa Elena Ophiolite alkaline spring, Costa Rica [7]; and 5) Coastal basin of Golfo Dulce, Costa Rica [8]. For 1), the publicly available metagenome-assemble genomes (MAGs) (https://gold.jgi.doe.gov/studies?id=Gs0114290) were also examined.

The metagenomics analysis pipeline consists of seven main stages and a preliminary Stage 0 for data and software preparation. In Stage 0, raw metagenomic reads in FASTQ format were downloaded from NCBI/SRA using *fastq-dump* from the SRA Toolkit [9]. When a sample was produced using paired-end sequencing, sample integrity was verified by confirming it contained the same number of forward and reverse reads. The average and standard deviation read count were then calculated. The algorithms and packages used in our pipeline are summarized below:

Stage	Function	Software	Ref	Link
0	Preliminary	*fastq-dump*	[9]	https://ncbi.github.io/sra-tools/fastq-dump.html
1	Quality control	*illlumina-utils*	[10]	https://github.com/merenlab/illumina-utils
2	Co-assembly	*MEGAHIT*<br>*Anvi'o*	[11]<br>[12]	https://github.com/voutcn/megahit<br>https://github.com/merenlab/anvio
3	Binning	*MaxBin*	[13]	https://downloads.jbei.org/data/microbial_communities/MaxBin/MaxBin.html
4	Refine bins	*CheckM*	[14]	http://ecogenomics.github.io/CheckM/
5	Functional annotation	*Prokka*	[15]	https://github.com/tseemann/prokka
6	Taxonomy classification of bins	*PhyloPhlAn*	[16]	https://bitbucket.org/nsegata/phylophlan/wiki/Home
7	Refine functional annotation of	*eggNOG-mapper*	[17]	https://github.com/jhcepas/eggnog-mapper

	methanotrophic MAGs			

For Stage 1 (quality control), high-quality reads were selected using *illlumina-utils* [10] with the Minoche [18] method using the command *iu-filter-quality-minoche* with the parameter *--ignore-deflines*. As the only exception, we used the method *iu-merge-pairs* for the metagenome from Serpentinite Springs of the Voltri Massif, Italy as the authors reported that the sequencing in this project yielded partially overlapped paired-end reads. In Stage 2 (co-assembly), pooled samples from the same environment were co-assembled following published methods [19–21]. Briefly, reads that passed quality control were co-assembled into contigs with *MEGAHIT* using the parameter *--min-contig-len* = 1000. Contigs produced by *MEGAHIT* [11] were subjected to refinement with *anvi'o* [12] three times using the parameter *--simplify-names* and setting *--min-len* to 1000, 1500, and 2500. In Stage 3 (binning), refined contigs and high-quality reads were binned with *MaxBin* [13] to produce MAGs (MAGs and bins refer to the same item in this pipeline). In Stage 4 (refine bins), the quality of MAGs was assessed with *CheckM* [14] and retained if they exceeded the quality standards defined in the Minimum Information about a Metagenome-Assembled Genome (MIMAG) for bacteria [22] for a medium-quality draft (completeness > 70% and contamination < 10%). MAGs of potential methanotrophs were selected according to the presence of methane oxidation genes (*pmoCAB* or *mmoXYZCDB*) in their genomes. In Stage 5 (functional annotation), MAGs were annotated with *Prokka* [15] using the parameters *–metagenome* and *--kingdom=Bacteria*. In Stage 6 (taxonomic classification), the amino acid sequences produced by *Prokka* were used as input for taxonomic characterization of MAGs using *PhyloPhlAn* [16] with parameters *-i* and *-t*. Only MAGs resulting in a taxonomic classification with high or medium confidence were selected for subsequent analyses. Finally, in Stage 7 (functional annotation refinement), all MAGs characterized as probable methanotrophic bacteria were subjected to a second, more comprehensive annotation procedures with *eggNOG-mapper* [17], in which KEGG Orthologs (KO), Gene Ontology (GO), and Clusters of Orthologous Groups (COGs) were assigned to genome features. The retrieved publicly available

assembled and binned MAGs of methanotrophic bacteria of Lake Washington, USA [4] were subjected to our metagenomics analysis pipeline from Stage 4 to Stage 7.

Geographical location of methanotroph genomes. The geographical coordinates of the origin for each sample were determined either from manual inspection of published reports (Table S1) or IMG/JGI [23]. When available, the exact coordinates of the sampling location were used to place genomes in the map. For nine genomes, the origin location could not be found using either method. The coordinates were plotted using the *maps* [24] package in *R*. The *position_jitter* parameters were set to $w = 3.1$and $h = 3.1$ to avoid overlapping of dots.

Genome-scale phylogenetic tree of genomes and MAGs. 59 methanotroph genomes and MAGs and one outgroup genome of the non-methanotrophic bacterium *Bacteroides ovatus* ATCC 8483 were used to reconstruct the phylogenetic tree with *PhyloPhlAn* [16] with parameter *-u* (*de novo* phylogenetic tree).

Incorporating metadata and nucleotide content into genome-scale phylogeny. The resultant phylogenetic tree (raw tree *1_proteomes_tree.nwk* available in GitHub repository) was imported to *R* using the *ape* package [25] and re-rooted to the outgroup genome of *B. ovatus* ATCC 8483. The outgroup genome was selected based on its close placement to known methanotrophs in the microbial tree of life [26]. Metadata of genomes and MAGs were also imported in order to assign features to each genome and to differentiate the seven methanotroph types. Methanotroph types were assigned using the *treeio* [27] *R* package. In the tree, number of coding sequences (CDSs) and distribution of GC and GC_3 content were plotted using the *R* packages *ggtree* [28] and *ggridges* [29]. GC and GC_3 compositions of CDSs were determined using the *gc* and *gc3* functions of the *seqinr* [30] *R* package. The standalone version of *EMBOSS* [31] was used to corroborate the GC and GC_3 content of each CDS of our interest. All the data were compiled and manually curated and are available in the file *1_QC_CH4.txt* in our GitHub repository.

Analysis of relative synonymous codon usage (RSCU). The frequency of individual codon usage per CDS normalized to the amino acid usage of its

corresponding protein was calculated as RSCU [32] using the function *uco* with parameter *index = rscu* from the *seqinr R* package. The equation used to calculate the RSCU is:

$$RSCU = \frac{O_{ij}}{\frac{\left[\sum_{j}^{n_i} O_{ij}\right] * 1}{n_i}} \qquad (1)$$

where O_{ij} is the occurrence of the jth codon for the ith amino acid and n_i the total number of synonymous codons coding for the ith amino acid. We considered a codon frequently used if RSCU ≥ 1.6, and rarely used if RSCU ≤ 0.6. Principal component analysis (PCA) was computed using the *R* function *prcomp* to identify CDSs that share similar preferences codon usage biases based on RSCU values for 59 codons (the conventional set of 64 codons excluding the two non-redundant codons for methionine and tryptophan, which have a fixed RSCU = 1.0, and the three stop codons).

Calculation of the codon adaptation index (CAI). The CAI [32] was used to analyze the codon usage of each CDS relative to a reference set of CDSs. Codon frequency was calculated for each CDS in each of the 67 isolate genomes and MAGs. Frequencies were calculated for a single reading frame of the CDS and only ~1% of all CDSs had length not divisible by three. The codon relative adaptiveness (*w*) was calculated as the frequency of a codon divided by the frequency of the synonymous codon with the highest frequency [32]. *w* values were used to compute the CAI for each codon using the *cai* function from the *seqinr R* package, using either the full set of CDSs in the genome (CAI_{genome}) or only ribosomal protein genes ($CAI_{ribosome}$). The percentile rank of each CDS within the distribution of $CAI_{genome}/CAI_{ribosome}$ was calculated.

Analysis of the effective number of codons (ENC). ENC [33] is a measure of CDS codon usage bias based on codon preference per amino acid and has been

applied recently to study genomes assembled from environmental samples [34, 35]. ENC values were computed for each CDS using the *chips* program from *EMBOSS* [31] based on Wright's equation [33]:

$$ENC = 2 + \frac{9}{\hat{F}_2} + \frac{1}{\hat{F}_3} + \frac{5}{\hat{F}_4} + \frac{3}{\hat{F}_6} \qquad (2)$$

where $\hat{F}_i$ is the codon homozygosity for the amino acids of degeneracy *i*. ENC as a function of GC_3 content was analyzed in all methanotrophs. A linear model relating ENC and GC_3 was fitted using the *stat_smooth* function from the *ggplot2* R package.

tRNA copy number. tRNA frequencies were analyzed only for isolate genomes where the total tRNA pool should be known. When available, the tRNA counts of the genomes were downloaded from the public databases of IMG/JGI and GtRNAdb [36, 37], otherwise they were computed with the local version of tRNAscan-SE 2.0 [38].

tRNA adaptation index (tAI). tAI was developed to estimate translation efficiency [39, 40]. The tAI was calculated for all CDSs in each genome using the *R* package *codonR* [40] with the parameter *sking* set to *1* (Prokaryote super kingdom) and the default *s* parameter for codon selection penalties. The tAI computation required the genomic tRNA counts (Table S2) and CDS codon frequencies, which were calculated using *CodonM*. Within-genome tAI percentile ranks were calculated for each CDS. The manually curated dataset containing the tAI data for our CDSs of interest can be found in the file *1_QC_V_manuallycurated.txt* in our GitHub repository.

Interaction network of codons and tRNAs. The interaction network was reconstructed for isolates of type Ia methanotrophs based on RSCU values for six CDS sets, tRNA copy numbers and codon-anticodon pairing rules. Four CDS sets (*pmoCAB*, *mmoXYZCDB*, *mxaFI* and *xoxF*) represented the methane oxidation metabolic module, one set comprised ribosomal protein genes, and one set comprised all the CDSs in each genome. The median RSCU of each codon for each

CDS set was computed from the distribution of RSCU values of all type Ia methanotrophs. The median copy number of each tRNA anticodon was calculated from all the copy numbers of all tRNA anticodons in type Ia methanotrophs. The tRNA anticodon matrix is shown in Fig. S4e. Standard codon-anticodon recognition rules [40] were used and are detailed in *0_wobble_pairing_rules.txt* available in our GitHub repository.

The integrated dataset was transformed into a network of sources (tRNA anticodons) and targets (CDS codons). The raw network matrix can be found in the file *RSCU_complete_network.txt* in GitHub. The matrix was imported to Cytoscape [41] and edited as shown in the Cytoscape file *1_Fig3D_net_cytoscape.cys*. The complete network containing all amino acids and codons is shown in Fig. S5a. A quantitative analysis was applied to the raw network matrix (*RSCU_complete_network.txt*). For each CDS, the number of accessible tRNA copies was calculated for a range of RSCU thresholds (e.g. for RSCU threshold = 0 each CDS can access every possible tRNA). This allowed the number of tRNA copies that a CDS can access as function of codon bias usage (as determined by RSCU) to be calculated. For each CDS, access to the tRNA pool can be measured in absolute term and relative to the tRNA pool available when compared with the access granted to other CDSs. The significance of the difference between two states (RSCU = 0 and RSCU = 2) was assessed with a Chi-square test, with accessible tRNA copies at RSCU = 0.0 as the expected value and at RSCU = 2.0 as the observed value. The test was applied only to CDSs of the methane oxidation metabolic module.

CDS amino acid composition. For each CDS, the codon exhibiting the highest median RSCU for each amino acid was selected. Methionine and tryptophan were excluded as they are each encoded by only one codon. The median and standard deviation were calculated from the distribution of RSCU values of each type of methanotrophs. The median and standard deviation of amino acid composition of each translated CDS of each operon was calculated using the distribution of the amino acid composition of each type of methanotrophs. A linear model (using the *lm*

function in *R*) was fitted to determine the relationship between codon preference and amino acid usage to serve as a proxy to identify selection for optimal codons at synonymous sites occupied by the most abundant amino acids.

Prebiotic amino acids analysis. The amino acid content of each protein in the metabolic module of all methanotrophs was calculated from its translated CDS. Amino acids were categorized as ‘cheap’/prebiotic (alanine, aspartic acid, glutamic acid, glycine, isoleucine, leucine, proline, serine, threonine, and valine) or ‘expensive’/modern amino acids [42–44]. A *t*-test was used to compute the statistical significance of the difference between modern and prebiotic amino acid composition of each protein in the metabolic module from type Ia methanotrophs. The sample for this test was the median amino acid composition (Fig. S5b).

Transcriptome analysis. Transcribed CDSs were analyzed by modifying thymine (T) for uracil (U) in all CDSs. Three publicly available type Ia methanotroph transcriptomic datasets (*Methylomicrobium buryatense* 5G [45], *Methylomicrobium alcaliphilum* 20Z [46] and *Methylobacter tundripaludum* 31/32 [47]) were used. Normalized mRNA abundance was obtained from each dataset as reported. The purine (A+G) and pyrimidine (T+C) content of each transcribed CDS was calculated. The purine and pyrimidine content of each transcriptome was calculated based on the ribonucleotide composition of each transcribed CDS multiplied by the transcript abundance, summed across all transcribed CDSs. The effect on transcriptome composition of removing a set of transcripts was calculated by subtracting the total transcribed CDS composition (transcribed CDS composition × transcript abundance) from the dataset and re-calculating the total ribonucleotide composition.

Elemental composition of transcribed CDSs. The carbon (C), hydrogen (H), oxygen (O) and nitrogen (N) composition of transcribed CDSs was calculated based on ribonucleotide molecular formulae (adenine $C_5H_5N_5$, guanine $C_5H_5N_5O$, cytosine $C_4H_5N_3O$, uracil $C_4H_4N_2O_2$) and normalized to the number of codons in each CDS. To provide statistical support for the observations of elemental composition bias in *pmoCAB* transcripts, the mean per-codon elemental content of 1,000 randomly selected combinations of three transcribed CDSs was calculated.

Correlation between transcript abundance and elemental composition. It has been recently proposed that highly expressed genes tend to decrease per-codon nitrogen requirements of their RNA transcripts [48, 49]. The relationship between elemental composition and mRNA abundance was investigated by computing the Pearson correlation coefficient with 95% confidence levels.

References

1. R Core Team. R: A language and environment for statistical computing. R Foundation for Statistical Computing, Vienna, Austria. 2017.

2. RStudio Team. RStudio: Integrated development for R. RStudio, Inc., Boston, MA. 2015.

3. Wickham, H. ggplot2: elegant graphics for data analysis. Springer-Verlag New York. 2016.

4. Oshkin, I. Y. et al. Methane-fed microbial microcosms show differential community dynamics and pinpoint taxa involved in communal response. *ISME J.* 2015; 9:1119–1129.

5. Brazelton, W. J. et al. Metagenomic identification of active methanogens and methanotrophs in serpentinite springs of the Voltri Massif, Italy. *PeerJ*. 2017; 5:e2945.

6. Kumaresan, D. et al. Aerobic proteobacterial methylotrophs in Movile Cave: genomic and metagenomic analyses. *Microbiome*. 2018; 6:1.

7. Crespo-Medina, M. et al. Methane dynamics in a tropical serpentinizing environment: the Santa Elena Ophiolite, Costa Rica. *Front Microbiol*. 2017; 8: 916.

8. Padilla, C. C. et al. Metagenomic binning recovers a transcriptionally active gammaproteobacterium linking methanotrophy to partial denitrification in an anoxic oxygen minimum zone. *Front Mar Sci*. 2017; 4:23.

9. Leinonen, R., Sugawara, H. & Shumway, M. The sequence read archive. *Nucleic Acids Res*. 2011; 39:D19–D21.

10. Eren, M. A., Vineis, J. H., Morrison, H. G. & Sogin, M. L. A Filtering method to generate high quality short reads using Illumina paired-end technology. *PLoS ONE*. 2013; 8:e66643.

11. Li, D. et al. MEGAHIT v1.0: A fast and scalable metagenome assembler driven by advanced methodologies and community practices. *Methods*. 2016; 102:3–11.

12. Eren, M. A. et al. Anvi'o: an advanced analysis and visualization platform for 'omics data. *PeerJ*. 20153;e1319.

13. Wu, Y.-W., Simmons, B. A. & Singer, S. W. MaxBin 2.0: an automated binning algorithm to recover genomes from multiple metagenomic datasets. *Bioinformatics*. 2016; 32:605–607.

14. Parks, D. H., Imelfort, M., Skennerton, C. T., Hugenholtz, P. & Tyson, G. W. CheckM: assessing the quality of microbial genomes recovered from isolates, single cells, and metagenomes. *Genome Res*. 2015; 25:1043–1055.

15. Seemann, T. Prokka: rapid prokaryotic genome annotation. *Bioinformatics*. 2014; 30:2068–2069.

16. Segata, N., Börnigen, D., Morgan, X. C. & Huttenhower, C. PhyloPhlAn is a new method for improved phylogenetic and taxonomic placement of microbes. *Nat Commun*. 2013; 4.

17. Huerta-Cepas, J. et al. Fast genome-wide functional annotation through orthology assignment by eggNOG-mapper. *Mol Biol Evol*. 2017; 34:2115–2122.

18. Minoche, A. E., Dohm, J. C. & Himmelbauer, H. Evaluation of genomic high-throughput sequencing data generated on Illumina HiSeq and Genome Analyzer systems. *Genome Biol*. 2011; 12:1–15.

19. Parks, D. H. et al. Recovery of nearly 8,000 metagenome-assembled genomes substantially expands the tree of life. *Nat Microbiol*. 2017; 2:1.

20. Stewart, R. D. et al. Assembly of 913 microbial genomes from metagenomic sequencing of the cow rumen. *Nat Commun*. 2018; 9:870.

21. Delmont, T. O. et al. Nitrogen-fixing populations of Planctomycetes and Proteobacteria are abundant in surface ocean metagenomes. *Nat Microbiol*. 2018; 3:804–813.

22. Bowers, R. M. et al. Minimum information about a single amplified genome (MISAG) and a metagenome-assembled genome (MIMAG) of bacteria and archaea. *Nat Biotechnol*. 2017; 35:725–731.

23. Markowitz, V. M. et al. IMG/M 4 version of the integrated metagenome comparative analysis system. *Nucleic Acids Res*. 2014; 42:D568–D573.

24. Becker, R. A., Wilks, A. R., Brownrigg, R., Minka, T. P. & Deckmyn, A. maps: draw geographical maps. R package version 3.3.0.2018.

25. Paradis, E., Claude, J. & Strimmer, K. APE: analyses of phylogenetics and evolution in R language. *Bioinformatics.* 2004; 20, 289–290.

26. Hug, L. A. et al. A new view of the tree of life. *Nat Microbiol*. 2016; 1:16048.

27. Yu, G. & Tsan-Yuk Lam, T. treeio: base classes and functions for phylogenetic tree input and output. R package version 1.2.2. 2018.

28. Yu, G., Smith, D. K., Zhu, H., Guan, Y. & Lam, T. ggtree: an R package for visualization and annotation of phylogenetic trees with their covariates and other associated data. *Methods Ecol Evol*. 2017; 8:28–36.

29. Wilke, C. O. ggridges: ridgeline plots in ‘ggplot2’. R package version 0.5.0.2018.

30. Charif, D. & Lobry, J. R. Biological and medical physics, biomedical engineering. 2007:207–232. doi:10.1007/978-3-540-35306-5_10

31. Rice, P., Longden, I. & Bleasby, A. EMBOSS: the European Molecular Biology Open Software Suite. *Trends Genet*. 2000; 16:276–277.

32. Sharp, P. M. & Li, W.-H. The codon adaptation index-a measure of directional synonymous codon usage bias, and its potential applications. *Nucleic Acids Res*. 1987; 15:1281–1295.

33. Wright, F. The ‘effective number of codons’ used in a gene. *Gene*. 1990; 87:23–29.

34. Alves, R. J., Minh, B., Urich, T., von Haeseler, A. & Schleper, C. Unifying the global phylogeny and environmental distribution of ammonia-oxidising archaea based on *amoA* genes. *Nat Commun*. 2018; 9:1517.

35. Zhalnina, K. et al. Dynamic root exudate chemistry and microbial substrate preferences drive patterns in rhizosphere microbial community assembly. *Nat Microbiol*. 2018; 3:470–480.

36. Chan, P. P. & Lowe, T. M. GtRNAdb: a database of transfer RNA genes detected in genomic sequence. *Nucleic Acids Res*. 2009; 37:D93–D97.

37. Chan, P. P. & Lowe, T. M. GtRNAdb 2.0: an expanded database of transfer RNA genes identified in complete and draft genomes. *Nucleic Acids Res*. 2016; 44:D184–D189.

38. Lowe, T. M. & Eddy, S. R. tRNAscan-SE: A program for improved detection of transfer RNA genes in genomic sequence. *Nucleic Acids Res*. 1997; 25:955–964.

39. dos Reis, M., Wernisch, L. & Savva, R. Unexpected correlations between gene expression and codon usage bias from microarray data for the whole *Escherichia coli* K-12 genome. *Nucleic Acids Res*. 2003; 31:6976-6985.

40. dos Reis, M., Savva, R. & Wernisch, L. Solving the riddle of codon usage preferences: a test for translational selection. *Nucleic Acids Res*. 2004; 32:5036–5044.

41. Shannon, P. et al. Cytoscape: a software environment for integrated models of biomolecular interaction networks. *Genome Res*. 2003; 13:2498–2504.

42. Li, N., Lv, J. & Niu, D.-K. Low contents of carbon and nitrogen in highly abundant proteins: evidence of selection for the economy of atomic composition. *J Mol Evol*. 2009; 68:248–255.

43. Longo, L. M., Lee, J. & Blaber, M. Simplified protein design biased for prebiotic amino acids yields a foldable, halophilic protein. *Proc Natl Acad Sci USA*. 2013; 110:2135–2139.

44. Blanco, L. P., Payne, B. L., Feyertag, F. & Alvarez-Ponce, D. Proteins of generalist and specialist pathogens differ in their amino acid composition. *Life Sci Alliance*. 2018; 1:e201800017.

45. Torre, A. et al. Genome-scale metabolic reconstructions and theoretical investigation of methane conversion in *Methylomicrobium buryatense* strain 5G(B1). *Microb Cell Fact*. 2015; 14:1–15.

46. Kalyuzhnaya, M. et al. Highly efficient methane biocatalysis revealed in a methanotrophic bacterium. *Nat Commun*. 2013; 4:2785.

47. Krause, S. et al. Lanthanide-dependent cross-feeding of methane-derived carbon is linked by microbial community interactions. *Proc Natl Acad Sci USA*. 2017; 114:358–363.

48. Seward, E. A. & Kelly, S. Dietary nitrogen alters codon bias and genome composition in parasitic microorganisms. *Genome Biol*. 2016; 17:226.

49. Seward, E. A. & Kelly, S. Selection-driven cost-efficiency optimization of transcripts modulates gene evolutionary rate in bacteria. *Genome Biol*. 2018; 19:102.
